# Combining individual patient data from randomized and non-randomized studies to predict real-world effectiveness of interventions

**DOI:** 10.1177/09622802221090759

**Published:** 2022-04-26

**Authors:** Michael Seo, Thomas PA Debray, Yann Ruffieux, Sandro Gsteiger, Sylwia Bujkiewicz, Axel Finckh, Matthias Egger, Orestis Efthimiou

**Affiliations:** 1 Institute of Social and Preventive Medicine, 27210 University of Bern, Bern, Switzerland; 2 Graduate School for Health Sciences, 27210 University of Bern, Bern, Switzerland; 3 Julius Center for Health Sciences and Primary Care, University Medical Center Utrecht, 8125 Utrecht University, Utrecht, The Netherlands; 4 Smart Data Analysis and Statistics B.V., Utrecht, The Netherlands; 5 Pharmaceuticals Division, Global Access, F. Hoffmann-La Roche, Basel, Switzerland; 6 Biostatistics Research Group, Department of Health Sciences, 4488 University of Leicester, Leicester, UK; 7 Division of Rheumatology, 30576 University Hospitals of Geneva, Geneva, Switzerland; 8 Population Health Sciences, Bristol Medical School, University of Bristol, Bristol, UK; 9 Department of Psychiatry, 6396 University of Oxford, Oxford, UK

**Keywords:** Real-world effectiveness, individual patient data, non-randomized studies, network meta-analysis, efficacy-effectiveness gap

## Abstract

Meta-analysis of randomized controlled trials is generally considered the most reliable source of estimates of relative treatment effects. However, in the last few years, there has been interest in using non-randomized studies to complement evidence from randomized controlled trials. Several meta-analytical models have been proposed to this end. Such models mainly focussed on estimating the average relative effects of interventions. In real-life clinical practice, when deciding on how to treat a patient, it might be of great interest to have personalized predictions of absolute outcomes under several available treatment options. This paper describes a general framework for developing models that combine individual patient data from randomized controlled trials and non-randomized study when aiming to predict outcomes for a set of competing medical interventions applied in real-world clinical settings. We also discuss methods for measuring the models’ performance to identify the optimal model to use in each setting. We focus on the case of continuous outcomes and illustrate our methods using a data set from rheumatoid arthritis, comprising patient-level data from three randomized controlled trials and two registries from Switzerland and Britain.

## Introduction

Randomized clinical trials (RCTs) and meta-analyses (MAs) of clinical trials are usually thought to be the most reliable source of evidence to evaluate the effects of medical interventions.^1^ However, RCTs are often carried out in specific settings and apply strict inclusion and exclusion criteria, which leads to results that may not be generalizable to routine practice.^2^ This situation leads to the so-called efficacy-effectiveness gap,^3,4^ where ‘efficacy’ refers to the performance of a medical intervention under experimental conditions, while ‘effectiveness’ relates to what is achieved in everyday clinical practice.

In the last few years, there has been growing interest in utilizing ‘real-world’ data from non-randomized studies (NRSs), to complement evidence from RCTs in medical decision-making, aiming to bridge the efficacy-effectiveness gap.^5–8^ The increased interest in NRSs was accompanied by the development of various meta-analytical modelling approaches,^9–12^ and by extending methods to network meta-analysis (NMA) and individual patient data (IPD) MA.^13–15^ So far, methods have primarily focussed on estimating relative treatment effects. When making (shared) decisions^16^ on how to treat patients in real-world settings, the prediction of (possibly several) outcomes under all competing treatments for individual patients or specific groups of patients is of great interest. In this context, jointly synthesizing IPD from RCTs and non-randomized, real-world studies in a prediction framework is promising. This idea was previously explored by Didden et al.,^17^ who aimed to predict the average, population-level, real-world effectiveness of a treatment pre-launch (i.e. before a treatment becomes available for clinical practice). However, to the best of our knowledge, there are no methods that combine patient-level data from multiple studies, randomized and observational, to provide personalized predictions for various treatments for patients treated in real-world settings.

We set out to fill this gap and propose a general prediction framework. We extend prediction modelling methods to an NMA setting^18^ to synthesize evidence on multiple interventions, utilizing IPD from randomized and NRSs. We propose a range of modelling approaches for predicting real-world outcomes and describe methods for selecting the optimal model to use. We focus on two-stage models, where at the first stage each study is analysed separately, and at the second stage the study-specific results are meta-analysed. Although one-stage approaches to IPD-MA and IPD-NMA are generally considered more flexible,^19^ a two-stage approach is often necessary for practice, due to data restrictions, e.g. when data reside on different servers. We illustrate how to implement our framework, using a real example in rheumatoid arthritis (RA), combining data from multiple trials and registries.

## A clinical example in RA

We used IPD from three RCTs and two NRS (based on disease registries) on patients diagnosed with RA, a chronic inflammatory disease characterized by progressive damage of joints.^20^ Several drugs can be prescribed for the treatment of RA. In this example, we focus on three: conventional synthetic disease-modifying anti-rheumatic drugs (DMARDs), combination therapy with rituximab (RTX) + DMARDs, and a combination of tocilizumab (TCZ) + DMARDs. Note that RTX and TCZ are also DMARDs, but they are biologic in contrast to conventional DMARDs such as methotrexate, which we refer to as DMARDs. The outcome of interest was the Disease Activity Score 28 (DAS28),^21^ which is continuous and ranges from 0 to 10. A lower score indicates lower disease activity.

**Figure 1.**
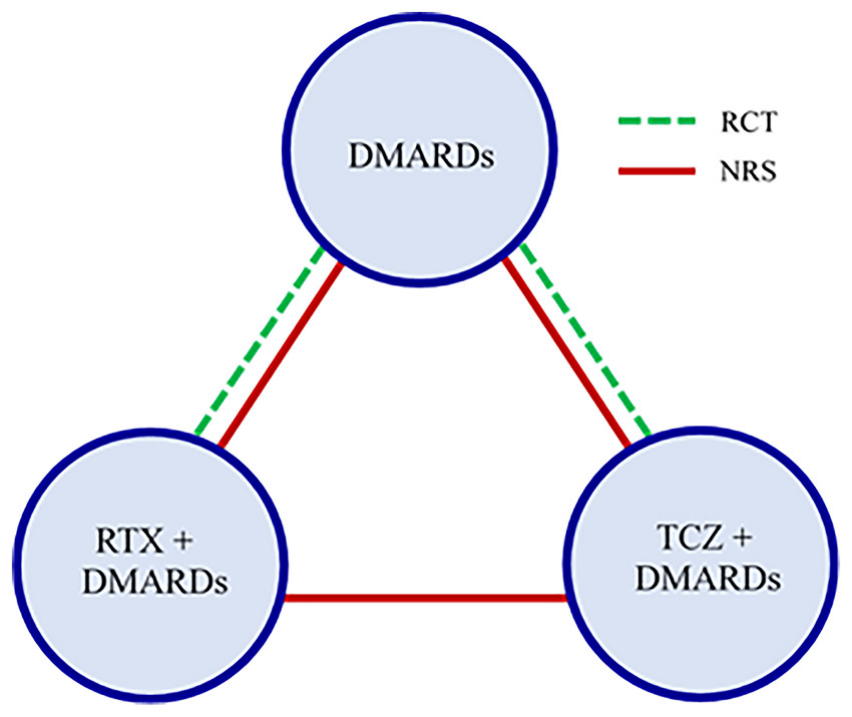
Network graph for the rheumatoid arthritis example. Green lines show RCTs and red lines show NRS. There were three two-armed RCTs; two of them compared TCZ + DMARDs vs DMARDs and one compared RTX + DMARDs vs DMARDs. There were two NRS, which included all three drugs. Abbreviations: DMARDs: Conventional disease-modifying anti-rheumatic drugs; RTX: rituximab; TCZ: tocilizumab; RCT: randomized controlled trial; NRS: non-randomized study.

One RCT (REFLEX) compared RTX + DMARDs versus DMARDs and included 517 patients. The other two RCTs (TOWARD and TOWARD2) compared TCZ + DMARDs versus DMARDs, and included 1216 and 791 patients, respectively. In all three RCTs, DAS28 was at baseline and after 6 months. For the two NRS, we used the measurement of DAS28 at 6 month (or the closest available measurement, within 3 months) after the start of the new drug. The British Society for Rheumatology Biologics Register in Rheumatoid Arthritis (BSRBR-RA) and the Swiss Clinical Quality Management in Rheumatoid Arthritis (SCQM) registries provided observational data.^22,23^ These included 2057 and 1069 patients, respectively. Established in 2001, BSRBR-RA is one of the largest studies looking at the long-term safety of new drugs prescribed for RA. It was launched when anti-tumor necrosis factor (TNF) therapies became available.^21^ Similarly, SCQM is a Swiss registry established in 1997 and allowed rheumatologists to follow their RA patients to improve outcomes.^22^ In addition to baseline DAS28, nine patient-level covariates were available from both RCTs and NRS at baseline: gender, age, disease duration, body mass index, baseline rheumatoid factor, number of previous DMARDs and anti-TNF agents, baseline health assessment questionnaire disability index, and baseline erythrocyte sedimentation rate (ESR). We provide additional details on the studies in Tables 1–3 of the Appendix. Figure 1 shows the network graph and overview of data sets.

## Methods

### Overview

Below, we propose a statistical framework to facilitate personalized medicine when IPD from NRS and RCTs are available. This framework can be used to build a multivariable model that predicts (absolute) patient-level, real-world outcomes for a range of different treatments for a given disease. We assume that the available data are IPD from a set of RCTs and NRS that compare two or more treatments for the same disease. The final product of our analyses will be a model where the input is a vector of patient-level covariates , and the output is the predicted outcome under each treatment . In what follows, we focus on the case of a continuous outcome and we limit our investigation to models with linear functions of the covariates. The model is as follows:

(1) $$y_{\mathrm{pred}}(x,t)=\alpha +\beta^{T}x\;+\gamma_{tA}^{T}x+\delta_{tA}$$

where *A* is a reference treatment and is a vector of patient-level covariates that may impact the outcome, possibly interacting with treatment. Without loss of generality, we assume all continuous covariates to be standardized, to facilitate model fitting. Next, is the predicted outcome under treatment *A* when ; encompasses the prognostic ability of the covariates; expresses the effect modification (i.e. treatment-covariate interactions) of treatment *t* versus *A*; and is the relative treatment effect *t* versus *A* for . We set and equal to zero. If a non-linear relationship is suspected, we can expand Equation (1) to include fractional polynomials or cubic-spline terms. Such extensions are straightforward within the framework described below but are beyond the scope of this paper.

We describe a range of meta-analytical two-stage approaches that can be used to estimate the parameters in the prediction model of Equation (1). First, we analyse each study separately. Second, we meta-analyse the study-specific estimates, to estimate all parameters needed for Equation (1).

We focus on Bayesian approaches because of their flexibility and ease of propagating uncertainty. We describe three generic approaches for building prediction models that combine IPD from studies of variable design.

- *Approach I*: We only use the IPD from a single NRS, i.e. without using MA.
- *Approach II*: We combine the IPD from all studies (NRS and RCTs) using a two-stage NMA, considering three different implementations. In approach IIa, we do not account for differences in study design. In approach IIb we employ shrinkage methods. In approach IIc, we further calibrate^24^ the intercept and main effects of covariates to target a specific patient group, using data from a single NRS that reflects this population.
- *Approach III*: We combine the IPD from NRS and RCTs using a two-stage NMA and adopt a weighting scheme to account for different study designs (IIIa). Again, we allow for re-calibrating the intercept term and main effects of covariates to target a specific patient group (IIIb).

As an additional, simplified method, we combine all studies as in IIa but we do not include any treatment-covariate interactions, i.e. we set . We call this Approach IV. After describing the various approaches in detail, we discuss ways of assessing their performance. Table 1 summarizes each approach, briefly describing the model and citing equations used.

**Table 1.** Overview of the different modelling approaches presented in this paper.

Approach	Description	Prediction for a new patient with covariates , treatment	Meta-analysis	in prediction model estimated from:	in prediction model estimated from:	Shrinkage at the first stage	Formulas used at the first stage	Formulas used at the second stage
I	We only use a single NRS.		**X**	A single NRS	A single NRS	✓	(2) and (3)	-
IIa	First stage: fit a model in each study separately.		✓	All RCTs and NRS	All RCTs and NRS	**X**	(3)	(5) or (6)
	Second stage: fit a design-naïve NMA.							
IIb	Same as IIa, but at first stage we use penalized estimation.		✓	All RCTs and NRS	All RCTs and NRS	✓	(2) and (3)	(5) or (6)
IIc	Same as IIb, but intercept and main effects are estimated from a single NRS.		✓	A single NRS	All RCTs and NRS	✓	(2) and (3)	(5) or (6)
IIIa	First stage: as per IIb		✓	All NRS	A combination of RCTs and NRS	✓	(2) and (3)	(7) and (8)
	Second stage: use weights according to study design.							
IIIb	Same as IIIa, but intercept and main effects are estimated from a single NRS.		✓	A single NRS	A combination of RCTs and NRS	✓	(2) and (3)	(7)
IV	Same as IIa, but without effect modifiers		✓	All RCTs and NRS	All RCTs and NRS	**X**	(9)	(10)

Abbreviations: RCT: randomized clinical trial. NRS: non-randomized study. NMA: network meta-analysis.

### Approach I: Analysis of a single study, reflecting the target population

Let us assume that our aim is to predict outcomes for a real-world population from which we have a representative sample, including patient-level data (e.g. from a specific registry). We can build a prediction model using these data alone (e.g. the model of Equation (1)) and disregard all other sources of information. This approach is relatively common in the literature, although (a) usual applications do not account for multiple interventions, and (b) these models are often based on IPD from a single RCT.

To enhance the predictive performance of the model, we can incorporate shrinkage in the model estimation. Shrinkage methods are known to improve prediction accuracy,^24,25^ while it has been recently shown that they can be useful in a MA setting, when aiming to estimate patient-level treatment effects.^26^ We are particularly interested in shrinking treatment-covariate interactions because information on effect modification is important, as it may substantially affect decision making.

There are many different frequentist as well as Bayesian shrinkage methods we could use. For example, for Bayesian shrinkage, we can penalize the coefficients of the effect modification (treatment-covariate interactions, i.e. parameters in Equation (1)) using a Laplace prior distribution. When the scale parameter of the Laplace prior distribution is assigned its own prior distribution, the model is often referred to as a Bayesian LASSO.^27,28^ The prior for effect modifiers of treatment *W* versus the reference *A* is given by:

(2) $$\pi (\gamma_{WA}|\lambda )=\overset{n_{cov}}{\underset{k=1}{\prod }}\frac{\lambda }{2}e^{-\lambda |\gamma_{k,WA}|}$$

where denotes the total number of covariates, is the regressor for effect modification of the *k* th covariate, and is the shrinkage parameter that controls how much estimates are shrunk to zero. There are several ways to select the shrinkage parameter, e.g. we can use -fold cross validation or choose the value via Bayes factor.^29^ We hereby chose to treat it as random and assign a non-informative hyperprior. Details on the hyperprior are given in the ‘Fitting the models’ section. Of note, Bayesian LASSO does not strictly perform variable selection, since covariates are not shrunk all the way to zero. As discussed above, we used penalization only for . Alternatively, we could also penalize prognostic factors, i.e. the parameters as well. For further discussion on possible extensions of this model we refer the reader to the Discussion section.

### Approach II: Design-naïve NMA

In this approach, we use all studies to fit a two-stage IPD NMA model.^30^ We disregard the information on study design, i.e. we treat RCTs and NRS the same. Thus, we use all studies to inform parameters , , in Equation (1). The parameter of this model, which denotes the predicted outcome for zero value of the covariates under the reference treatment A, will only be informed by studies that included this treatment. This means that the choice of reference treatment is potentially important (while the choice of reference is arbitrary in the standard NMA model.^18^) An obvious choice *A* is the treatment that is given to most patients across the available data sets.

Let us assume that patient *i* was included in the study *j*, received treatment , and that for this patient the observed outcome of interest was , measured on a continuous scale. Also, we assume that for this patient we have covariates. Let us also assume that the study had only two treatment arms, *W* and *A* (the reference). Under these definitions, the first stage model is as follows:

(3) $$\begin{array}{rl} & y_{ij}∼N(m_{ij},\sigma_{j}^{2})\\ & m_{ij}=a_{j}+b_{j}^{T}x_{ij}+c_{j,t_{ij}A}^{T}\;x_{ij}+d_{\;j,t_{ij}A}\\ & \sigma_{j}^{2},\;a_{j},\;b_{j},\;c_{j,WA},d_{\;j,WA}\;∼\;(\mathrm{vague}\;\mathrm{prior}\;\mathrm{distributions}) \end{array}$$

where we set ; denotes the expected outcome (linear predictor); is the study-specific variance of the outcome; is the study-specific intercept; is the vector of study-specific regression coefficients for the main effects of the covariates; is the vector of coefficients for effect modification (i.e. treatment-covariate interaction) for study *j* and treatment *W* relative to *A*; and is the treatment effect at , for study *j* and treatment *W* relative to *A*. Prior distributions for these parameters are described in more detail in the ‘Fitting the models’ section. Thus, at the first stage of the model we estimate and the corresponding variance-covariance matrix .

These estimates contribute to the likelihood of the second stage of the MA, via a multivariate random-effects model:

(4) $$\begin{array}{rl} & {\hat{\theta }}_{j}∼N(\xi_{j},\;{\hat{S}}_{j})\\ & \xi_{j}∼N((\alpha ,\beta ,\gamma_{WA},\;\delta_{WA}),\;\Sigma )\\ & \left(\alpha ,\beta ,\gamma_{WA},\delta_{WA},\;\Sigma )∼\;(\mathrm{vague}\;\mathrm{prior}\;\mathrm{distributions}\right) \end{array}$$

where is a between-study (i.e. random effects) variance-covariance matrix. Note that when using the IPD NMA model, we are typically interested in estimating relative effects (i.e. , ), while is usually set to be study-specific and is considered a nuisance parameter. Here, however, we want to predict absolute outcomes; thus, we also require estimates of , . The estimation of might be difficult, especially for a few studies or many covariates. In this case, we may simplify the model by assuming , , and to be common (fixed), i.e. independent of the study.

Furthermore, a typical assumption in NMA is that the between-study variance of the random effects is the same across treatment contrasts.^18^ Then, the second stage model can be written as follows:

(5) $$\begin{array}{rl} {\hat{\theta }}_{j} & ∼N((\alpha ,\beta ,\gamma_{WA},\;\theta_{\;j,WA}^{\left(\delta \right)}),\;{\hat{S}}_{j})\\ & \theta_{\;j,WA}^{\left(\delta \right)}∼N(\delta_{WA},\;\tau^{2})\\ \left(\alpha ,\beta ,\gamma_{WA},\delta_{WA},\;\tau^{2}\right) & ∼\;(\mathrm{vague}\;\mathrm{prior}\;\mathrm{distributions}) \end{array}$$

For multi-arm studies and for studies that do not include reference treatment *A*, the models need adaptation; we provide details in Section 2 of the Appendix.

Again, it may be difficult to estimate heterogeneity of the treatment effect when only a few studies are available. This is the case for the RA example described in the ‘A clinical example in RA’ section. One solution would be to use external information to create an informative prior distribution for . For example, for the case of binary outcomes, Turner et al.^31^ proposed empirical distributions that can be used in Bayesian MAs. A further simplification would be to assume common :

(6) $$\begin{array}{rl} {\hat{\theta }}_{j} & ∼N((\alpha ,\beta ,\gamma_{WA},\;\delta_{WA}),\;{\hat{S}}_{j})\\ \left(\alpha ,\beta ,\gamma_{WA},\delta_{WA}\right) & ∼\;(\mathrm{vague}\;\mathrm{prior}\;\mathrm{distributions}) \end{array}$$

We call Approach IIa the combination of Equation (3) and Equation (5) or (6) to estimate the parameters of Equation (1). Approach IIb is an extension of IIa, where we use the shrinkage method (i.e. Bayesian LASSO) at the first stage.

Lastly, we can further extend Approach IIb by calibrating the intercept and main effects of covariates to target a specific real-world population. A motivation for this is that data sampled from the patient population of interest might be the best source of evidence for predicting the reference treatment outcome. Conversely, estimates of the intercept term obtained from RCTs might be less representative for the target population because RCT patients are selected and because RCTs are performed in highly controlled settings.

In detail, we use Approach I to estimate , using only data obtained from a study that reflects the target population, and Approach IIb, using all data to estimate , . We then use Equation (1) to make predictions for individual patients. We call this Approach IIc. To summarize, in Approach IIc we use study-specific estimates of , and pooled relative effects, i.e. , . Note that we fit the full model within each study separately, and then pool all parameters (including all main effects and interactions) in the second stage of the model across studies. Table 1 summarizes all the different flavours of Approach II.

### Approach III: Design-adjusted analysis

For Approach III, when aggregating parameters at the second stage, we use a weighting scheme for studies of a different design.^15^ Specifically, we weight the first-stage estimates of relative treatment effects from Approach IIb according to the corresponding study's design.

In this approach, the variance of the estimates of treatment effects and effect modification obtained from NRS *j* (i.e. the variance of , ) is inflated after dividing by a factor , with . By doing so, we effectively decrease the impact of NRS in the estimation of all relative treatment effects. Setting corresponds to completely disregarding estimates of relative treatment effects obtained from NRS.

This approach's motivation is that RCTs are usually thought to be the most reliable sources of information for relative treatment effects because randomization helps us avoid issues related to confounding. Moreover, since we aim to predict outcomes for real-world populations, estimates for the model's intercept and the main effects of the covariates are aggregated only using NRS. The second stage model is as follows:

(7) $$\begin{array}{rl} & ({\hat{c}}_{j,WA},{\hat{d}}_{\;j,WA})∼\{\begin{array}{c} \;N\left((\gamma_{WA},\;\delta_{WA}),\frac{{\hat{S}}_{j}}{w_{j}}\right),\;\;\mathrm{if}\;\mathrm{study}\;j\;\mathrm{is}\;\mathrm{an}\;\mathrm{NRS}\\ N((\gamma_{WA},\;\delta_{WA}),{\hat{S}}_{j}),\;\;\mathrm{if}\;\mathrm{study}\;j\;\mathrm{is}\;\mathrm{an}\;\mathrm{RCT} \end{array}\\ & \left(\gamma_{WA},\delta_{WA})∼\;(\mathrm{vague}\;\mathrm{prior}\;\mathrm{distributions}\right) \end{array}$$

and

(8) $$\begin{array}{rl} \left({\hat{a}}_{j},{\hat{b}}_{j}\right) & ∼N((\alpha ,\beta ),\;{\hat{S}}_{j}),\;\;\;\mathrm{if}\;\mathrm{study}\;j\;\mathrm{is}\;a\;\mathrm{NRS}\\ \;(\alpha ,\beta ) & ∼\;(\mathrm{vague}\;\mathrm{prior}\;\mathrm{distributions}) \end{array}$$

For choosing the weights we can consider e.g. study quality, or how similar is the healthcare system of an NRS from a specific country as compared to that of a target population;^15^ in practice, a range of values can be used, and model performance measures can be assessed (see next section) to decide on the optimal weights. See also the Discussion section for some additional considerations regarding weights. We call this Approach IIIa. Finally, instead of aggregating NRS to estimate study intercept and main effects, we can calibrate to target a population as in Approach IIc. We call this approach IIIb.

### Approach IV: Design-naïve NMA without effect modifiers

For Approach IV, we fit the similar design-naïve NMA, but without including effect modifiers (i.e. setting ). This may serve as a sensitivity analysis, and by comparing its results with the previous methods we might obtain insights on the extent of heterogeneity of treatment effects. The linear predictor is now

(9) $$m_{ij}=a_{j}+b_{j}^{T}x_{ij}+d_{j,t_{ij}A}$$

and the multivariate MA is

(10) $$\begin{array}{rl} {\hat{\theta }}_{j} & ∼N(\psi_{j},\;{\hat{S}}_{j})\\ \psi_{j} & ∼N((\alpha ,\beta ,\;\delta_{WA}),\;\Sigma )\\ \left(\alpha ,\beta ,\;\delta_{WA},\;\Sigma \right) & ∼\;(\mathrm{vague}\;\mathrm{prior}\;\mathrm{distributions}) \end{array}$$

$$\mathrm{where}\;\hat{\theta_{j}}=(a_{j},b_{j},d_{j,t_{ij}A})$$

### Assessing the performance of the prediction models

A common practice to measure a prediction model's performance for a continuous outcome is through mean squared error (MSE) and bias:

(11) $$\mathrm{MSE}=\frac{1}{N}\underset{i,j}{\sum }\;({\hat{y}}_{ij}-y_{ij}{)}^{2},\;\;\mathrm{Bias}=\frac{1}{N}\underset{i,j}{\sum }\;({\hat{y}}_{ij}-y_{ij})\;$$

where *N* is the total number of patients in a given study of interest, is the predicted outcome using equation (1) and is the observed outcome. Values close to zero indicate good performance. Note that since the focus is on predicting real-world outcomes, metrics should be evaluated on NRS data.

We can also calculate the coefficient of determination (R-squared) to evaluate a model's performance for a given study of interest. We do that by using only and from each model:

(12) $$R^{2}=1-\frac{SS_{\mathrm{res}}}{SS_{\mathrm{tot}}}\;$$

where is the total sum of squares, is the average observed outcome, and is the residual sum of squares. We can also fit a regression line to inspect the agreement between observed and predicted patient outcomes, i.e.

(13) $$y_{ij}=\eta_{0}+\overset{K}{\underset{k=1}{\sum }}\;\eta_{k}{\hat{y}}_{ij}I(t_{ij}=k)$$

where *K* is the total number of treatments and is an indicator function that equals 1 if the treatment assigned to a patient *i* in study *j* was *k*, and 0 otherwise. This ‘calibration line’ compares the observed with the predicted outcome across different treatment groups. Having close to 0 and , …, values close to 1 indicate good performance of the prediction models.

The performance measures in equations (11), (12), and (13) inform us of our models’ overall predictive ability. However, when deciding on how to treat, we are mainly interested in selecting the best treatment for each patient. Thus, a prediction model that would be useful for deciding between competing treatments should also be well calibrated in terms of estimated treatment benefit. To achieve this, we reshape the calibration line using the predicted benefit (i.e. the difference between predicted outcomes under different treatments). For networks with treatments , , , … for each of the competing models we fit the line

(14) $$\begin{array}{rl} y_{ij}= & \kappa_{0}+\kappa_{1}{\hat{y}}_{ij,1}+\kappa_{2}({\hat{y}}_{ij,2}-{\hat{y}}_{ij,1})I(t_{ij}=2)\\ & \;+\kappa_{3}({\hat{y}}_{ij,3}-{\hat{y}}_{ij,1})I(t_{ij}=3)+\ldots \end{array}$$

where , , , … are the predicted outcomes of a patient under treatments , , … respectively, under this model. Similarly, having close to 0 and , , , … values close to 1 indicate good performance of the prediction models.

### Cross validation

For each competing model, we can calculate the performance metrics discussed above using internal as well as an internal–external cross validation.^32,33^

For internal validation, we use all available data to develop the models. Then, given that we aim to predict real-world outcomes, we use the NRS to test all models, i.e. to compare predictions with observations using the measures described in the previous section. To correct for optimism,^24^ we can calculate optimism-corrected performance using bootstrapping as discussed by Steyerberg.^24^ More details on this procedure are given in Section 2.2 of the Appendix. An internal validation procedure will inform us on which model performs best for each specific setting, provided we stratify the procedure by study.

For internal-external cross-validation, we exclude one NRS from the analysis and use the rest of the data to train the models. We then use the left-out NRS to make predictions and compare them with observations. Finally, we cycle through all available NRS. To follow this approach, we need data from multiple NRS to be available. If this is not the case, we may split the single NRS data in a meaningful non-random way, e.g. by clinic, region, or any other clustering variable in our data set. This internal-external validation procedure can potentially provide insights on which model might perform better in new settings.

The internal–external cross-validation via leave-one-study-out cannot be combined with Approach I, as this analysis only uses a single study to train the model. In this case, we can instead use all NRS except the left-out one to develop the model. For instance, for the RA case study, we only used the British registry to train the model and the Swiss registry to make predictions, and vice versa. Moreover, for Approach IIc and IIIb, when using internal-external cross-validation, we use all NRS except the left-out to estimate intercept and main effects of covariates, i.e. we do not use RCTs.

## Implementation details

Below we provide some details on implementing the described models in the RA example.

### Standardization of covariates

To use penalized estimation in the analysis of study *j* we need to standardize variables. This is necessary to ensure that penalization is equally applied to all regressors.^25^ Standardizing means transforming each covariate in each study by subtracting the study-specific mean from the covariate and dividing the result with the covariate's study-specific standard deviation. However, standardizing makes it difficult to MA results from multiple studies coherently, given that in each study the covariates are transformed differently. Thus, before aggregating the first stage results at the second stage, we need to revert coefficients to their natural scale. The mathematical details of how to do this are provided in Section 2.3 of the Appendix.

### Imputation of missing data

Gelman et al.^34^ recommended the following approach to handle missing data in Bayesian analyses via Markov Chain Monte Carlo (MCMC): (a) create *m* multiply imputed data sets; (b) analyse each imputed data set separately; (c) combine the *m* posterior draws, i.e. by mixing the corresponding draws.

We use R package mice^35^ to impute the missing covariates to create multiply imputed data sets.^36^ When imputing we used information from covariates, treatment, covariate-treatment interactions, and outcomes, but we did not impute the missing outcomes for model development. Imputation was done in each study separately using the method of predictive mean matching.

### Fitting the models

All analyses were carried out in R^37^ using rjags.^38^ For all models, DMARDs were the reference treatment. When fitting the first stage models, we used 20 imputed data sets and ran 3 chains of 10,000 iterations each, with 1000 burn-in. For the second stage models, we ran 3 chains of 200,000 iterations with 20,000 burn-in. We assessed convergence using the Gelman and Rubin diagnostics.^39^

For all models, we used a vague prior distribution for the standard deviation of continuous outcomes (). For regression parameters of stage one (i.e. , , , and ), we used a distribution. When applying Bayesian LASSO in stage one, a vague prior distribution was placed on the scale parameter for Laplace prior (). We tested the sensitivity of results to the selection of prior distribution on . For Approach III, we used weights 0.25 and 0.5.

For calculating optimism-corrected performance, 200 bootstrap samples were drawn. The R codes used for fitting all models are available at https://github.com/MikeJSeo/phd/tree/master/ra.

## Results

The parameter estimates from the first-stage analysis for each study are shown in Tables 4, 5, 6, and 7 in the Appendix. There were some important differences between RCTs and NRS, especially in the case of the Swiss registry. Τhe estimated intercept term for this study was much smaller than for the RCTs, probably owing to the fact that the Swiss registry had the lowest average DAS28 score; see Table 3 in the Appendix. Moreover, the average relative treatment effects of the biologic treatments versus DMARDs estimated in the registries were smaller than in the RCTs. These differences might raise concerns about the direct applicability of the RCT findings in patients found in real-world settings. We might hypothesize that these differences were due to residual confounding, model misspecification (e.g. omission of non-linear or interaction terms), or due to the more general ‘efficacy-effectiveness gap’ described in the Introduction.

Furthermore, there was only very weak evidence of heterogeneous treatment effects (i.e. effect modification) across all studies. Results from the second-stage analysis are shown in Table 8 of the Appendix. As expected, given the first stage results, methods that gave more weight to the RCTs in the estimation of relative effects provided larger effects. Next, we assessed all models’ performance using the measures described in the ‘Assessing the performance of the prediction models’ section and following the two cross-validation approaches described in the ‘Cross validation’ section.

**Figure 2.**
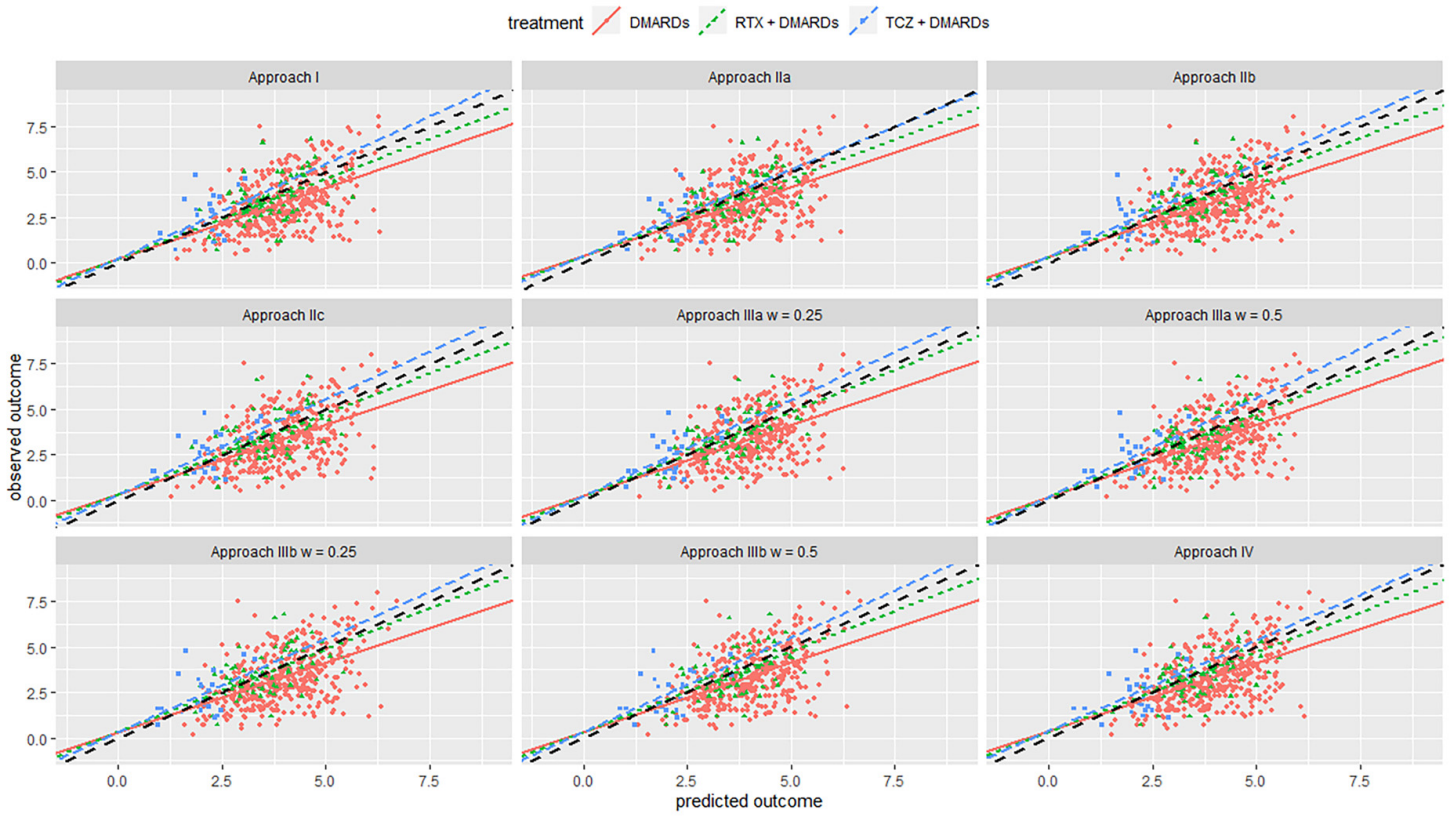
Calibration plot from internal–external validation, for the Swiss registry as the external data set. Black line is line of perfect calibration. Red line is the slope for DMARDs; green line is for RTX + DMARDs; blue line is for TCZ + DMARDs. Each dot represents one patient. Abbreviations: DMARDs: Disease-modifying anti-rheumatic drugs; RTX: rituximab; TCZ: tocilizumab.

We first discuss the results from the internal validation. In Table 2, we give results in terms of MSE and Bias, and in Table 3 results for the calibration lines and R-squared value. Figures 1 and 2 in the Appendix show the calibration plots. Overall, we saw that for most approaches the bias was rather small in clinical terms. MSE was around 1.5 for all models; to bring it to the same scale as the outcome we can calculate the root of MSE, i.e. 1.22, deemed to be small-to-moderate in clinical terms. R-squared was moderate (around 0.30 for all approaches). Moreover, we see that most models had similar performance. For the Swiss registry, Approach I performed slightly better for most measures of performance. For the British registry, approach IIIa was overall the best, especially in measures of calibration. However, differences with other methods, including Approach I, were not very pronounced. This showed that, when predicting real-world outcomes, utilizing data from multiple studies and using advanced analysis methods brought no benefit to the Swiss and small benefits to the British registry.

**Table 2.** Internal validation results of bias and MSE for different approaches.

Data set	Treatment arms	Performance metric	Approach I	Approach IIa	Approach IIb	Approach IIc	Approach IIIa *w* = 0.25	Approach IIIa *w* = 0.5	Approach IIIb *w* = 0.25	Approach IIIb *w* = 0.5	Approach IV
SCQM	All arms	MSE	1.44	1.54	1.59	1.46	1.54	1.53	1.47	1.47	1.62
		Bias	0.10	0.22	0.25	0.05	0.18	0.18	0.04	0.05	0.27
	DMARDs	MSE	1.55	1.66	1.72	1.55	1.65	1.64	1.55	1.55	1.75
		Bias	0.11	0.27	0.32	0.11	0.25	0.24	0.11	0.11	0.35
	RTX + DMARDs	MSE	0.94	1.05	1.03	0.98	1.04	1.04	0.99	0.98	1.04
		Bias	0.11	0.12	0.10	−0.09	0.05	0.10	−0.13	−0.06	0.06
	TCZ + DMARDs	MSE	1.13	0.99	1.10	1.44	1.24	1.21	1.54	1.51	1.13
		Bias	−0.16	−0.24	−0.36	−0.58	−0.47	−0.44	−0.66	−0.63	−0.35
BSRBR-RA	All arms	MSE	1.39	1.43	1.43	1.45	1.42	1.41	1.44	1.44	1.45
		Bias	0.13	0.11	0.07	0.19	0.09	0.05	0.18	0.20	0.05
	DMARDs	MSE	1.43	1.40	1.38	1.43	1.30	1.42	1.43	1.43	1.38
		Bias	0.16	0.08	0.06	0.16	−0.03	0.08	0.16	0.16	0.02
	RTX + DMARDs	MSE	1.28	1.28	1.30	1.28	1.82	1.30	1.27	1.29	1.34
		Bias	0.11	0.09	0.04	0.14	0.13	0.01	0.14	0.19	0.00
	TCZ + DMARDs	MSE	1.66	1.97	1.98	2.10	1.98	1.80	2.00	2.00	2.02
		Bias	0.16	0.30	0.25	0.45	−0.30	0.13	0.38	0.40	0.31

Abbreviations: MSE: mean squared error; DMARDs: disease-modifying anti-rheumatic drugs; RTX: rituximab; TCZ: tocilizumab.

**Table 3.** Internal validation results of the calibration lines and R-squared for different approaches.

Data set	Performance metric	Approach I	Approach IIa	Approach IIb	Approach IIc	Approach IIIa w = 0.25	Approach IIIa w = 0.5	Approach IIIb w = 0.25	Approach IIIb w = 0.5	Approach IV
SCQM	Calibration slope for outcome	= 0.00 = 0.97 = 0.97 = 1.06	= 0.43 = 0.81 = 0.85 = 0.92	= 0.46 = 0.79 = 0.85 = 0.95	= 0.06 = 0.95 = 1.01 = 1.24	= 0.31 = 0.85 = 0.90 = 1.05	= 0.26 = 0.86 = 0.90 = 1.07	= 0.09 = 0.94 = 1.01 = 1.26	= 0.08 = 0.95 = 1.00 = 1.25	= 0.48 = 0.78 = 0.85 = 0.93
	Calibration slope for benefit	= -0.01 = 0.98 = 1.10 = 0.80	= 0.43 = 0.81 = 0.57 = 0.66	= 0.43 = 0.80 = 0.50 = 0.58	= 0.00 = 0.97 = 0.68 = 0.59	= 0.26 = 0.86 = 0.59 = 0.58	= 0.22 = 0.87 = 0.66 = 0.59	= 0.01 = 0.96 = 0.64 = 0.56	= 0.01 = 0.96 = 0.71 = 0.57	= 0.43 = 0.79 = 0.40 = 0.56
	R squared	0.29	0.24	0.22	0.28	0.24	0.25	0.28	0.28	0.20
BSRBR-RA	Calibration slope for outcome	= -0.46 = 1.07 = 1.08 = 1.09	= 0.29 = 0.92 = 0.91 = 0.78	= 0.27 = 0.93 = 0.93 = 0.79	= -0.07 = 0.99 = 0.98 = 0.85	= 0.05 = 0.97 = 0.99 = 0.91	= -0.03 = 0.99 = 1.01 = 0.94	= -0.03 = 0.98 = 0.98 = 0.86	= -0.08 = 0.99 = 0.97 = 0.87	= -0.36 = 0.92 = 0.91 = 0.75
	Calibration slope for benefit	= -0.43 = 1.07 = 1.04 = 1.05	= 0.26 = 0.93 = 0.98 = 1.14	= 0.24 = 0.94 = 1.01 = 1.13	= -0.14 = 1.00 = 0.99 = 1.18	= -0.02 = 0.99 = 0.96 = 1.06	= -0.08 = 1.01 = 1.06 = 1.07	= -0.09 = 0.99 = 0.98 = 1.14	= -0.10 = 0.99 = 1.07 = 1.15	= -0.33 = 0.92 = 0.90 = 1.16
	R squared	0.39	0.37	0.37	0.36	0.38	0.38	0.37	0.37	0.36

These follow from linear regressions of the observed vs. predicted, as noted in equations (13) and (14). Subscript 0 refers to the intercept, 1 refers to DMARDs, 2 refers to RTX + DMARDs, and 3 refers to TCZ + DMARDs. Abbreviations: DMARDs: disease-modifying anti-rheumatic drugs; RTX: rituximab; TCZ: tocilizumab.

**Figure 3.**
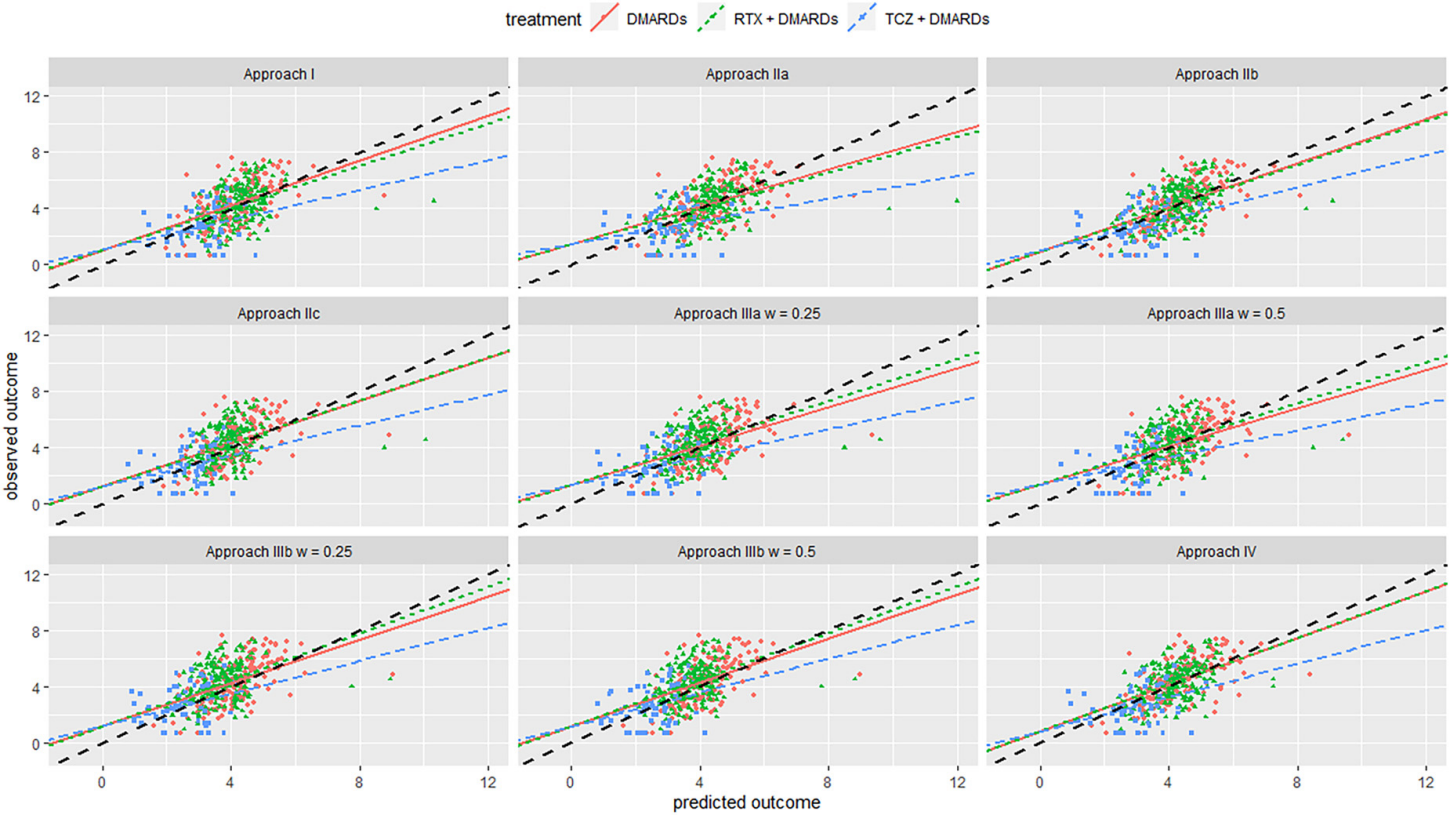
Calibration plot from internal-external validation, for the British registry as the external data set. Black line is line of perfect calibration. Red line is slope for DMARDs; green line is for RTX + DMARDs; the blue line is for TCZ + DMARDs. Each dot represents one patient. Abbreviations: DMARDs: Disease-modifying anti-rheumatic drugs; RTX: rituximab; TCZ: tocilizumab.

Next, we discuss results from the internal–external validation. This procedure gave us some insight into the models’ expected performance when applied in new settings. Results are summarized in Tables 4, 5, and Figures 2, 3. Overall, calibration lines’ intercepts were far from zero for most models, and R-squared values were quite low, indicating low performance. Removing Swiss registry data from the training set and using it for validation resulted in models IIa, IIb, and IIIb performing best. Likewise, when we left the British registry out, we saw that model IIb and IV performed best Approaches IIb and IV gave almost the same results and were overall the best for both registries. They had the lowest overall MSE, relatively higher R-squared value and lower bias for all treatment arms and were slightly better calibrated for both the absolute outcome and treatment benefit compared to the other models. This showed that utilizing information from RCTs can help us better predict real-world outcomes compared to using only data from NRS. It also showed that in this data set there was little evidence of an effect modification. Thus, if we aim to make predictions about patients in a new setting (e.g. another country), for which no data are currently available, we would recommend Approaches IIb or IV. See also the Discussion section on additional considerations regarding generalizability.

**Table 4.** Internal–external validation results for MSE and bias, for difference approaches.

Left-out data set	Treatment arms	Performance metric	Approach I	Approach IIa	Approach IIb	Approach IIc	Approach IIIa *w* = 0.25	Approach IIIa *w* = 0.5	Approach IIIb *w* = 0.25	Approach IIIb *w* = 0.5	Approach IV
SCQM	All arms	MSE	1.85	1.74	1.84	1.85	1.87	1.86	1.84	1.84	1.86
		Bias	0.51	0.42	0.48	0.48	0.50	0.51	0.47	0.48	0.50
	DMARDs	MSE	2.03	1.91	2.03	2.03	2.06	2.05	2.03	2.03	2.06
		Bias	0.61	0.52	0.61	0.61	0.65	0.64	0.61	0.61	0.63
	RTX + DMARDs	MSE	1.08	1.07	1.04	1.06	1.04	1.05	1.04	1.05	1.04
		Bias	0.20	0.10	0.10	0.08	0.02	0.09	−0.01	0.07	0.08
	TCZ + DMARDs	MSE	1.10	1.02	1.13	1.20	1.12	1.13	1.15	1.16	1.11
		Bias	−0.32	−0.29	−0.40	−0.46	−0.37	−0.39	−0.41	−0.42	−0.38
BSRBR-RA	All arms	MSE	1.67	1.72	1.55	1.71	1.74	1.73	1.79	1.76	1.52
		Bias	−0.05	0.11	0.02	−0.24	−0.17	−0.09	−0.38	−0.35	−0.02
	DMARDs	MSE	1.57	1.48	1.41	1.57	1.58	1.60	1.57	1.57	1.42
		Bias	−0.19	0.07	0.00	−0.19	0.05	0.11	−0.19	−0.19	−0.04
	RTX + DMARDs	MSE	1.67	1.81	1.53	1.80	1.86	1.80	1.97	1.90	1.45
		Bias	−0.04	0.08	−0.03	−0.35	−0.39	−0.29	−0.59	−0.54	−0.11
	TCZ + DMARDs	MSE	1.91	2.06	2.03	1.77	1.83	1.84	1.81	1.81	2.08
		Bias	0.34	0.29	0.25	−0.05	−0.03	0.03	−0.19	−0.18	0.32
Overall	All arms	MSE	1.76	1.73	1.70	1.78	1.81	1.80	1.82	1.80	1.70
		Bias	0.25	0.27	0.27	0.14	0.19	0.23	0.08	0.09	0.26
	DMARDs	MSE	1.90	1.78	1.84	1.90	1.92	1.92	1.90	1.90	1.87
		Bias	0.37	0.38	0.43	0.37	0.47	0.48	0.37	0.37	0.43
	RTX + DMARDs	MSE	1.51	1.62	1.40	1.60	1.64	1.60	1.72	1.67	1.34
		Bias	0.02	0.09	0.00	−0.23	−0.28	−0.18	−0.44	−0.38	−0.06
	TCZ + DMARDs	MSE	1.67	1.75	1.76	1.60	1.62	1.63	1.62	1.61	1.79
		Bias	0.14	0.12	0.06	−0.17	−0.13	−0.09	−0.25	−0.25	0.11

Abbreviations: MSE: mean squared error; DMARDs: disease-modifying anti-rheumatic drugs; RTX: Rituximab; TCZ: Tocilizumab.

**Table 5.** Internal–external validation results of the calibration lines and R-squared for different approaches.

Left-out Data set	Performance metric	Approach I	Approach IIa	Approach IIb	Approach IIc	Approach IIIa *w* = 0.25	Approach IIIa *w* = 0.5	Approach IIIb *w* = 0.25	Approach IIIb *w* = 0.5	Approach IV
SCQM	Calibration slope for outcome	= 0.25 = 0.78 = 0.88 = 1.04	= 0.40 = 0.76 = 0.86 = 0.96	= 0.35 = 0.76 = 0.88 = 1.02	= 0.34 = 0.76 = 0.88 = 1.05	= 0.23 = 0.78 = 0.93 = 1.05	= 0.18 = 0.79 = 0.92 = 1.08	= 0.33 = 0.76 = 0.91 = 1.03	= 0.32 = 0.76 = 0.89 = 1.04	= 0.35 = 0.75 = 0.88 = 1.03
	Calibration slope for benefit	= 0.22 = 0.79 = 0.23 = 0.45	= 0.35 = 0.78 = 0.29 = 0.53	= 0.27 = 0.78 = 0.23 = 0.46	= 0.24 = 0.78 = 0.20 = 0.43	= 0.14 = 0.80 = 0.19 = 0.44	= 0.09 = 0.82 = 0.21 = 0.44	= 0.23 = 0.79 = 0.19 = 0.44	= 0.23 = 0.79 = 0.20 = 0.44	= 0.25 = 0.78 = 0.20 = 0.44
	R squared	0.09	0.14	0.09	0.09	0.08	0.09	0.09	0.09	0.08
BSRBR-RA	Calibration slope for outcome	= 1.07 = 0.80 = 0.75 = 0.53	= 1.47 = 0.66 = 0.64 = 0.40	= 0.97 = 0.79 = 0.77 = 0.56	= 1.23 = 0.76 = 0.77 = 0.55	= 1.35 = 0.69 = 0.75 = 0.50	= 1.36 = 0.68 = 0.72 = 0.48	= 1.21 = 0.77 = 0.82 = 0.58	= 1.15 = 0.78 = 0.83 = 0.60	= 0.81 = 0.83 = 0.83 = 0.60
	Calibration slope for benefit	= 1.11 = 0.75 = 0.59 = 1.32	= 1.10 = 0.70 = 0.36 = 0.98	= 0.87 = 0.78 = 0.59 = 1.08	= 1.02 = 0.78 = 0.47 = 1.03	= 1.17 = 0.72 = 0.38 = 0.95	= 1.23 = 0.69 = 0.39 = 0.95	= 1.00 = 0.80 = 0.45 = 0.98	= 0.98 = 0.81 = 0.48 = 0.99	= 0.84 = 0.81 = 0.75 = 1.20
	R squared	0.27	0.25	0.32	0.25	0.23	0.24	0.21	0.23	0.33

These follow from linear regressions of the observed vs. predicted, as noted in equations (13) and (14). Subscript 0 refers to the intercept, 1 refers to DMARDs, 2 refers to RTX + DMARDs, and 3 refers to TCZ + DMARDs. Abbreviations: DMARDs: disease-modifying anti-rheumatic drugs; RTX: rituximab; TCZ: tocilizumab.

**Figure 4.**
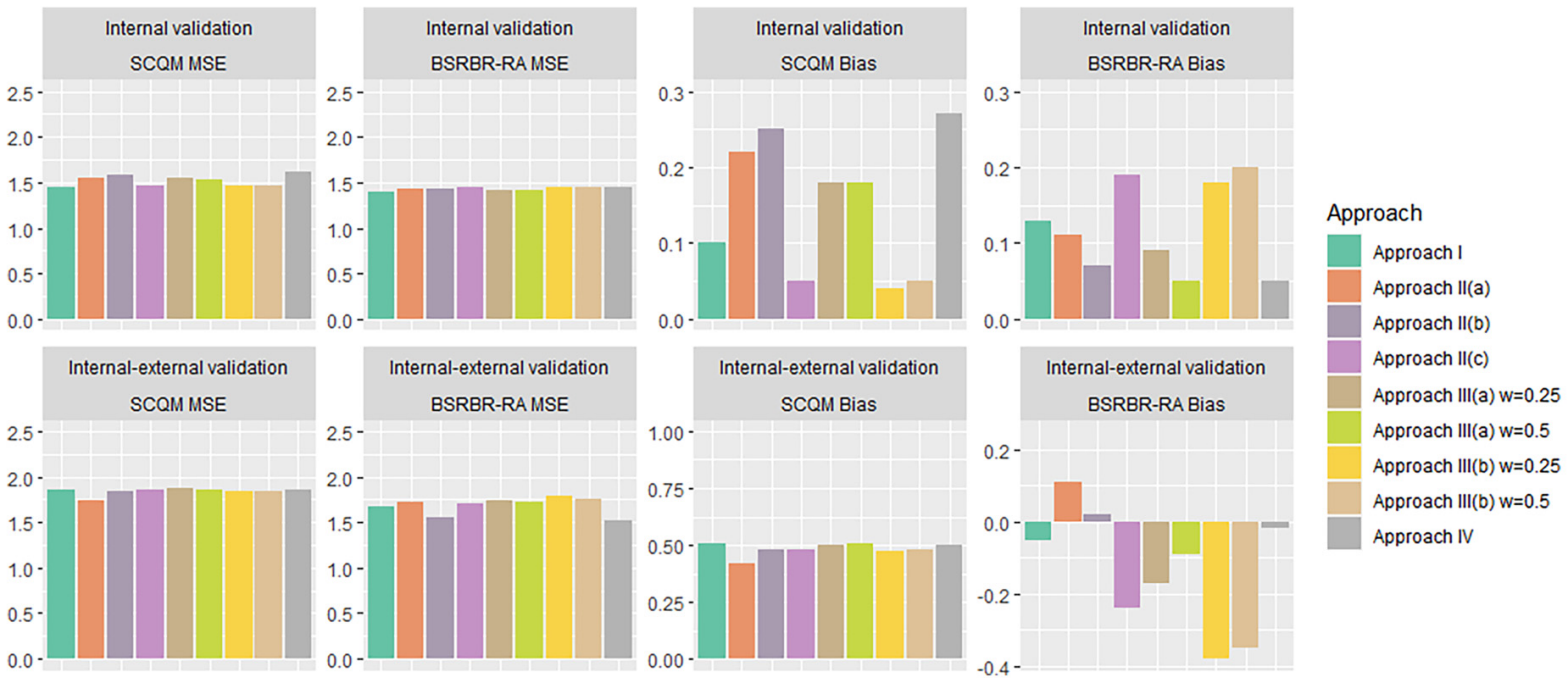
Bar plot summarizing MSE and bias calculated through an internal (top row) and internal–external (bottom row) validation, for the Swiss and British registry (SCQM and BSRBR-RA respectively). For internal–external validation, the labelled study is used as the target data set. Abbreviations: MSE: Mean squared error; DMARDs: Disease-modifying anti-rheumatic drugs; RTX: rituximab; TCZ: tocilizumab.

Figure 4 summarizes the MSE and bias results for both internal and internal-external validation. In Table 9 of the Appendix we show results after correcting for optimism. We saw that Approach I had slightly larger optimism, as compared to other methods. However, these optimism-corrected results did not materially change the conclusion drawn from the main analyses. This is because optimism was generally quite small for all models. This was expected, since the sample size was big, the outcome was (approximately) continuous, and we only used few predictors. Finally, results did not materially change when using different prior distributions for .

## Discussion

This paper presents a general framework for developing methods to predict real-world outcomes for a range of different treatment options. We focussed on continuous outcomes and proposed various prediction modelling approaches and methods for assessing their performance. Our models were based on two-stage IPD NMA and utilized both randomized and observational data.

We used a data set of patients with RA obtained from three RCTs and two registries to illustrate our methods. For this example, we developed six meta-analytical models and a simpler model (i.e. Approach I). The latter only utilized data from a single registry at a time. After fitting each model, we assessed its internal and internal–external performance while adjusting for optimism. In these validations, we compared observed outcomes versus predictions for all registry patients and calculated bias, MSE, and R-squared. We also fitted calibration lines, both for the overall outcome as well as for treatment benefit.

For our example, internal validation showed that Approach I was among the best performing approaches for both the Swiss and the British registry. Thus, for this particular example, the incorporation of RCT data and advanced meta-analytical modelling brought small benefit in terms of predictive ability for patients found in these two registries. Conversely, the internal–external cross-validation procedure identified Approaches IIb and IV as the best-performing ones. This suggested that these two might be the best for patients found in a completely new setting among all developed models. However, we should keep in mind that to establish the generalizability of a model, we would need data from multiple real-world settings to be available. In case of important heterogeneity (i.e. when there are great differences across different real-world settings), it will be tough to make predictions about patients found in new settings. This is one general limitation of all proposed methods.

Another important limitation of our approach is the need for IPD from multiple sources. IPD is generally hard to obtain at the MA level. However, new initiatives have been recently launched, such as YODA (https://yoda.yale.edu/), Vivli (https://vivli.org/), and Clinical Study Data Request (https://www.clinicalstudydatarequestcom/), aiming to promote large-scale IPD sharing. Moreover, our approach requires a connected network of interventions. In practical applications, the available NRSs may not include all interventions of interest (despite forming a closed network). In such cases, although we can still use some of the described methods to make predictions for outcomes under the missing treatments, there is no way to assess the goodness of these predictions for these particular settings. Another limitation is that we only discussed the case of comparative studies, i.e. studies that included more than one treatment. A generalization of the methods to include single-arm studies might be of interest in a follow-up project.

An additional limitation is that the proposed linear model (with only two-way interactions between treatment and covariate) might be too simple to predict accurately in the real-world. If a non-linear relationship is suspected, we could consider models that include fractional polynomials or cubic splines. Alternative approaches include tree-based methods.^40–42^ However, we did not explore them in detail.

In Approach III, we used fixed weighting factors . We arbitrarily used values of 0.25 and 0.5 for these factors. Sensitivity analysis showed that using different values did not alter much the performance of the model. Alternatively, we could have used flexible weighting, e.g. by assigning a prior distribution to , treating the weight as a random variable.^15^ This is similar to the ‘power prior’ approach.^43,44^ We fitted a model that used a modification of the power prior approach, called normalized power prior^45,46^ in the RA data set. In this model, the parameter estimates from NRS are down-weighted according to whether they agree with the RCT estimates. In the case of a large disagreement, there is more down-weighting. We found that the weighting factors decreased all the way down to zero for the RA data set, negating all information from the NRS. For large sample data sets the RCTs and NRS are likely to disagree (i.e. large discrepancies between the mean and variance of RCTs and NRS), which will lead to pronounced down-weighting.^47^ Thus, we did not show models based on the power prior in this paper; however, this might be an interesting area for future development. Also, we could assign weights to different model parameters, e.g. the intercept term, if deemed appropriate; we did not pursue this further in this paper. Another limitation of our methods is that it may often be difficult to use calibration measures (i.e. calibration intercept and slope for predicted outcome and predicted benefit) to compare the performance between competing models. It is possible that some models yield accurate outcome predictions (i.e. prognosis) but not of treatment benefit, and vice versa. Selecting an appropriate model will then strongly depend on the context in which the model should be used.

Moreover, we could have explored approaches where penalization also applies to prognostic factors and not only to effect modifiers, and also other penalization methods. Also, we only discussed two-stage meta-analytical approaches; however, making these methods one-stage is straightforward. Another interesting idea suggested by one of our anonymous reviewers would be to include study design as yet another predictor in the second-stage model. However, it would be difficult to implement in our data set, which only includes two NRS; we leave this idea for future research.

In this paper, we used a simple regression adjustment for estimating treatment effects from the observational studies. Alternative methods such as propensity score matching^48^ or inverse probability of treatment weighting (IPTW) could be used when the assumptions behind the regression adjustment seem implausible. We explored the use of IPTW and preliminary results showed a similar performance to the models based on regression adjustment. Thus, we decided to leave this for future work. Note, however, that no method for causal inference guarantees unbiased estimation of treatment effects from the observational studies. In our framework, when these estimates are biased, models that use this information (such as Approach IIa or IIb) are expected to perform worse, and thus not be selected at the end as the final model. Also, note that all methods for causal inference are limited by unmeasured confounding.

Furthermore, in practical applications, we often face the problem of ‘systematically missing’ predictors, i.e. when a predictor is missing for all individuals within particular studies in an IPD MA. There have been methods proposed for imputing such predictors, based on the missing at random assumption.^49^ There is also an R package micemd,^50^ which implements some of these imputation methods. This however requires all data to be on the same server; in our experience, this cannot always be achieved, especially when data from different studies are owned by different for-profit companies. In such cases, researchers may only get access to data via the companies’ private servers, without being able to merge data sets from different studies.

Finally, we note that our methods need to be extended to cover the case of binary and time-to-event outcomes. This might be challenging, especially when assessing the predictive performance of different approaches using calibration for treatment benefit. For example, the so-called c-statistic for benefit was recently proposed^51^ and might be used to this end; however, future research is needed to investigate this.

To summarize, this is the first paper to propose and compare two-stage meta-analytical IPD models for predicting the real-world effectiveness of interventions to the best of our knowledge. The gain in predictive performance from combining RCTs and NRS was modest in our clinical example. Nevertheless, the illustration of different modelling approaches and the considerations regarding different cross-validation methods that we provide may be valuable to inform future studies aiming to predict real-world outcomes of competing interventions.

## Supplemental Material

sj-docx-1-smm-10.1177_09622802221090759 - Supplemental material for Combining individual patient data from randomized and non-randomized studies to predict real-world effectiveness of interventions Supplemental material, sj-docx-1-smm-10.1177_09622802221090759 for Combining individual patient data from randomized and non-randomized studies to predict real-world effectiveness of interventions by Michael Seo, Thomas PA Debray, Yann Ruffieux, Sandro Gsteiger, Sylwia Bujkiewicz, Axel Finckh, Matthias Egger and Orestis Efthimiou in Statistical Methods in Medical Research

## References

[1] Evans D . Hierarchy of evidence: a framework for ranking evidence evaluating healthcare interventions. J Clin Nurs 2003; 12: 77–84. 12519253 10.1046/j.1365-2702.2003.00662.x

[2] Revicki DA Frank L . Pharmacoeconomic evaluation in the real world. Effectiveness versus efficacy studies. Pharmacoeconomics 1999; 15: 423–434. 10537960 10.2165/00019053-199915050-00001

[3] Eichler H-G Abadie E Breckenridge A , et al. Bridging the efficacy-effectiveness gap: a regulator’s perspective on addressing variability of drug response. Nat Rev Drug Discov 2011; 10: 495–506. 21720406 10.1038/nrd3501

[4] Nordon C Karcher H Groenwold RHH , et al. The ‘efficacy-effectiveness gap’: historical background and current conceptualization. Value Health 2016; 19: 75–81. 26797239 10.1016/j.jval.2015.09.2938

[5] Reeves BC Higgins JPT Ramsay C , et al. An introduction to methodological issues when including non-randomised studies in systematic reviews on the effects of interventions. Res Synth Methods 2013; 4: 1–11. 26053535 10.1002/jrsm.1068

[6] Schünemann HJ Tugwell P Reeves BC , et al. Non-randomized studies as a source of complementary, sequential or replacement evidence for randomized controlled trials in systematic reviews on the effects of interventions. Res Synth Methods 2013; 4: 49–62. 26053539 10.1002/jrsm.1078

[7] Concato J Shah N Horwitz RI . Randomized, controlled trials, observational studies, and the hierarchy of research designs. N Engl J Med 2000; 342: 1887–1892. 10861325 10.1056/NEJM200006223422507 PMC1557642

[8] Sarri G Patorno E Yuan H , et al. Framework for the synthesis of non-randomised studies and randomised controlled trials: a guidance on conducting a systematic review and meta-analysis for healthcare decision making. BMJ EBM 2020; **27**: 109–119. 10.1136/bmjebm-2020-111493 PMC8961747 33298465

[9] Turner RM Spiegelhalter DJ Smith GCS , et al. Bias modelling in evidence synthesis. J R Stat Soc Ser A Stat Soc 2009; 172: 21–47. 10.1111/j.1467-985X.2008.00547.x PMC2667303 19381328

[10] Ferguson J Alvarez-Iglesias A Newell J , et al. Joint incorporation of randomised and observational evidence in estimating treatment effects. Stat Methods Med Res 2019; 28: 235–247. 28745132 10.1177/0962280217720854

[11] Verde PE Ohmann C . Combining randomized and non-randomized evidence in clinical research: a review of methods and applications. Res Synth Methods 2015; 6: 45–62. 26035469 10.1002/jrsm.1122

[12] Verde PE . A bias-corrected meta-analysis model for combining, studies of different types and quality. Biometrical Journal 2020; **63**: 406–422. Epub ahead of print 30 September 2020. DOI: 10.1002/bimj.201900376. 32996196

[13] Cameron C Fireman B Hutton B , et al. Network meta-analysis incorporating randomized controlled trials and non-randomized comparative cohort studies for assessing the safety and effectiveness of medical treatments: challenges and opportunities. Syst Rev 2015; 4: 147–147. 26537988 10.1186/s13643-015-0133-0 PMC4634799

[14] Schmitz S Adams R Walsh C . Incorporating data from various trial designs into a mixed treatment comparison model. Stat Med 2013; 32: 2935–2949. 23440610 10.1002/sim.5764

[15] Efthimiou O Mavridis D Debray TPA , et al. Combining randomized and non-randomized evidence in network meta-analysis. Stat Med 2017; 36: 1210–1226. 28083901 10.1002/sim.7223

[16] Barry MJ Edgman-Levitan S . Shared decision making — the pinnacle of patient-centered care. N Engl J Med 2012; 366: 780–781. 22375967 10.1056/NEJMp1109283

[17] Didden E-M Ruffieux Y Hummel N , et al. Prediction of real-world drug effectiveness prelaunch: case study in rheumatoid arthritis. Med Decis Making 2018; 38: 719–729. 30074882 10.1177/0272989X18775975

[18] Efthimiou O Debray TPA Valkenhoef G van , et al. Getreal in network meta-analysis: a review of the methodology. Res Synth Methods 2016; 7: 236–263. 26754852 10.1002/jrsm.1195

[19] Debray TPA Moons KGM van Valkenhoef G , et al. Get real in individual participant data (IPD) meta-analysis: a review of the methodology. Res Synth Methods 2015; 6: 293–309. 26287812 10.1002/jrsm.1160 PMC5042043

[20] Grassi W De Angelis R Lamanna G , et al. The clinical features of rheumatoid arthritis. Eur J Radiol 1998; 27: S18–S24. 9652497 10.1016/s0720-048x(98)00038-2

[21] Fransen J Stucki G van Riel PLCM . Rheumatoid arthritis measures: disease activity score (DAS), disease activity score-28 (DAS28), rapid assessment of disease activity in rheumatology (RADAR), and rheumatoid arthritis disease activity Index (RADAI). Arthritis Care Res (Hoboken) 2003; 49: S214–S224.

[22] Dennison EM Packham J Hyrich K . The BSRBR-RA at 15 years. Rheumatology (Oxford) 2016; 55: 2093–2095. 27012687 10.1093/rheumatology/kew053 PMC5061081

[23] Langenegger T Fransen J Forster A , et al. Klinisches qualitätsmanagement bei der rheumatoiden arthritis. Zeitschrift für Rheumatologie 2001; 60: 333–341. 11759233 10.1007/s003930170033

[24] Steyerberg EW . Clinical prediction models. 2nd Ed. New York: Springer, 2019.

[25] Hastie T Tibshirani R Friedman J . The elements of statistical learning : data mining, inference, and prediction. New York: Springer, 2001: 43–99.

[26] Seo M White IR Furukawa TA , et al. Comparing methods for estimating patient-specific treatment effects in individual patient data meta-analysis. Stat Med 2021; 40: 1553–1573. 33368415 10.1002/sim.8859 PMC7898845

[27] Park T Casella G . The Bayesian lasso. J Am Stat Assoc 2008; 103: 681–686.

[28] O’Hara RB Sillanpää MJ . A review of Bayesian variable selection methods: what, how and which. Bayesian Anal 2009; 4: 85–117.

[29] Lykou A Ntzoufras I . On Bayesian lasso variable selection and the specification of the shrinkage parameter. Stat Comput 2013; 23: 361–390.

[30] Debray TP Schuit E Efthimiou O , et al. An overview of methods for network meta-analysis using individual participant data: when do benefits arise? Stat Methods Med Res 2018; 27: 1351–1364. 27487843 10.1177/0962280216660741

[31] Turner RM Jackson D Wei Y , et al. Predictive distributions for between-study heterogeneity and simple methods for their application in Bayesian meta-analysis. Stat Med 2015; 34: 984–998. 25475839 10.1002/sim.6381 PMC4383649

[32] Steyerberg EW Harrell FE Jr. Prediction models need appropriate internal, internal–external, and external validation. J Clin Epidemiol 2016; 69: 245–247. 25981519 10.1016/j.jclinepi.2015.04.005 PMC5578404

[33] Debray TPA Moons KGM Ahmed I , et al. A framework for developing, implementing, and evaluating clinical prediction models in an individual participant data meta-analysis. Stat Med 2013; 32: 3158–3180. 23307585 10.1002/sim.5732

[34] Gelman A Carlin JB Stern HS , et al. Bayesian Data analysis. 2nd ed. New York: Chapman and Hall/CRC, 2004.

[35] van Buuren S Groothuis-Oudshoorn K . Mice: multivariate imputation by chained equations in R. J Stat Softw 2011; 45: 67.

[36] Zhou X Reiter JP . A note on Bayesian inference after multiple imputation. Am Stat 2010; 64: 159–163.

[37] Core Team R . R: a language and environment for statistical computing. Vienna, Austria: R Foundation for Statistical Computing, https://www.R-project.org/ (2018 accessed 12 October 2021).

[38] Plummer M . Rjags: bayesian graphical models using MCMC, https://CRAN.R-project.org/package=rjags (2019).

[39] Gelman A Rubin DB . Inference from iterative simulation using multiple sequences. Stat Sci 1992; 7: 457–472.

[40] Li X Dusseldorp E Meulman JJ . A flexible approach to identify interaction effects between moderators in meta-analysis. Res Synth Methods 2019; 10: 134–152. 30511514 10.1002/jrsm.1334 PMC6590644

[41] Fokkema M Smits N Zeileis A , et al. Detecting treatment-subgroup interactions in clustered data with generalized linear mixed-effects model trees. Behav Res Methods 2018; 50: 2016–2034. 29071652 10.3758/s13428-017-0971-x

[42] Seibold H Zeileis A Hothorn T . Individual treatment effect prediction for amyotrophic lateral sclerosis patients. Stat Methods Med Res 2018; 27: 3104–3125. 29298618 10.1177/0962280217693034

[43] Ibrahim JG Chen M-H . Power prior distributions for regression models. Statist Sci 2000; 15: 46–60.

[44] Ibrahim JG Chen M-H Gwon Y , et al. The power prior: theory and applications. Stat Med 2015; 34: 3724–3749. 26346180 10.1002/sim.6728 PMC4626399

[45] Duan Y Ye K Smith EP . Evaluating water quality using power priors to incorporate historical information. Environmetrics 2006; 17: 95–106.

[46] Neuenschwander B Branson M Spiegelhalter DJ . A note on the power prior. Stat Med 2009; 28: 3562–3566. 19735071 10.1002/sim.3722

[47] Neelon B James A Malley O . Bayesian Analysis using power priors with application to pediatric quality of care. *J Biom Biostat* 2010; **1**: 1–9. Epub ahead of print 1 January 2010. DOI: 10.4172/2155-6180.1000103.

[48] Rosenbaum PR and Rubin DB. The central role of the propensity score in observational studies for causal effects. *Biometrika* 1983; **70**: 41–55.

[49] Audigier V White I Jolani S , et al. Multiple imputation for multilevel data with continuous and binary variables. *Stat Sci* 2018; **33**: 160–183. Epub ahead of print 2018. DOI: 10.1214/18-STS646.

[50] Audigier V , Resche-Rigon M. *micemd: multiple Imputation by Chained Equations with Multilevel Data*, https://CRAN.R-project.org/package=micemd (2018). 10.1177/0962280216666564 PMC5496677 27647809

[51] van Klaveren D Steyerberg EW Serruys PW , et al. The proposed ‘concordance-statistic for benefit’ provided a useful metric when modeling heterogeneous treatment effects. J Clin Epidemiol 2018; 94: 59–68. 29132832 10.1016/j.jclinepi.2017.10.021 PMC7448760

