# Supplementary material for: Combining individual patient data from randomized and non-randomized studies to predict real-world effectiveness of interventions
Source: Stat Methods Med Res. 2022 Apr 26;31(7):1355–73. doi: 10.1177/09622802221090759 (PMC9251754; doi:10.1177/09622802221090759)

**Combining individual patient data from randomized and non-randomized studies to predict real-world effectiveness of interventions**

Michael Seo^1,2 *^, Thomas P.A. Debray^3,4^, Yann Ruffieux^1^, Sandro Gsteiger^5^, Sylwia Bujkiewicz^6^, Axel Finckh^7^, Matthias Egger^1,8^, Orestis Efthimiou^1,9^

*^1^ Institute of Social and Preventive Medicine, University of Bern, Bern, Switzerland*

*^2^ Graduate School for Health Sciences, University of Bern, Bern, Switzerland*

*^3^ Julius Center for Health Sciences and Primary Care, University Medical Center Utrecht, Utrecht University, Utrecht, The Netherlands*

*^4^ Smart Data Analysis and Statistics, Utrecht, The Netherlands*

*^5^ Global Access, F. Hoffmann-La Roche, Pharmaceuticals Division, Basel, Switzerland*

*^6^ Biostatistics Research Group, Department of Health Sciences, University of Leicester, Leicester, United Kingdom*

*^7^ Division of Rheumatology, University Hospitals of Geneva, Geneva, Switzerland*

*^8^ Population Health Sciences, Bristol Medical School, University of Bristol, Bristol, United Kingdom*

*^9^ Department of Psychiatry, University of Oxford, Oxford, United Kingdom*

** corresponding author*

# Characteristics of rheumatoid arthritis datasets

**Table 1**: Overview of the different datasets. RCT: randomized controlled trial; NRS: non-randomized study; DMARDs: disease-modifying anti-rheumatic drugs; RTX: Rituximab; TCZ: Tocilizumab.

| **Design** | **RCT** | | | **NRS** | |
| --- | --- | --- | --- | --- | --- |
| **Name** | **REFLEX** | **TOWARD** | **TOWARD2** | **BSRBR-RA** | **SCQM** |
| **Treatment 1  (# of patients)** | DMARDs (209) | DMARDs (414) | DMARDs (392) | DMARDs (1169) | DMARDs (874) |
| **Treatment 2  (# of patients)** | RTX + DMARDs (308) | TCZ + DMARDs (802) | TCZ + DMARDs (399) | RTX + DMARDs (629) | RTX + DMARDs (135) |
| **Treatment 3  (# of patients)** |  |  |  | TCZ + DMARDs (259) | TCZ + DMARDs (60) |

**Table 2**: Number of patients with missing data on the outcome (DAS28) and covariates across different datasets. DAS28: Disease Activity Score 28; BMI: Body mass index; DMARD: Disease-modifying anti-rheumatic Drug; anti-TNF: anti-tumor necrosis factor; HAQ: health assessment questionnaire; ESR: erythrocyte sedimentation rate.

| **Variables** | **REFLEX (Total: 517)** | **TOWARD (Total: 1216)** | **TOWARD2 (Total: 791)** | **BSRBR-RA (Total: 2057)** | **SCQM  (Total: 1069)** |
| --- | --- | --- | --- | --- | --- |
| **DAS28** | 128 | 176 | 265 | 1076 | 171 |
| **Female** | 0 | 0 | 0 | 0 | 0 |
| **Age** | 0 | 0 | 0 | 0 | 0 |
| **Disease duration** | 0 | 1 | 0 | 21 | 49 |
| **BMI** | 0 | 3 | 7 | 490 | 345 |
| **Baseline rheumatoid factor** | 0 | 0 | 0 | 178 | 42 |
| **Number of previous DMARDs/anti-TNFs** | 0 | 0 | 0 | 0 | 0 |
| **Baseline HAQ disability index** | 1 | 11 | 47 | 476 | 239 |
| **Baseline ESR** | 10 | 0 | 5 | 302 | 71 |
| **Baseline DAS28** | 10 | 1 | 13 | 310 | 82 |

**Table 3**: Baseline characteristics of patients enrolled in the randomized clinical trials and registries. Numbers in parentheses are standard deviations. For binary outcomes, the proportion is reported. DAS28: Disease Activity Score 28; BMI: Body mass index; DMARD: Disease-modifying anti-rheumatic Drug; anti-TNF: anti-tumor necrosis factor; HAQ: health assessment questionnaire; ESR: erythrocyte sedimentation rate

| **Variables** | **REFLEX** | **TOWARD** | **TOWARD2** | **BSRBR-RA** | **SCQM** |
| --- | --- | --- | --- | --- | --- |
| **DAS28** | 4.90 (1.59) | 4.06 (1.69) | 3.93 (1.65) | 4.18 (1.51) | 3.41 (1.44) |
| **Female** | 0.81 | 0.82 | 0.83 | 0.76 | 0.74 |
| **Age** | 52.50 (12.40) | 53.19 (12.76) | 52.33 (12.11) | 59.37 (12.22) | 56.00 (13.62) |
| **Disease duration** | 11.90 (8.05) | 9.80 (8.88) | 9.10 (8.18) | 11.73 (10.00) | 6.88 (8.43) |
| **BMI** | 28.78 (7.08) | 27.73 (6.31) | 27.74 (6.44) | 28.16 (10.96) | 25.58 (4.83) |
| **Baseline rheumatoid factor** | 0.78 | 0.77 | 0.82 | 0.64 | 0.75 |
| **Number of previous DMARDs/anti-TNFs** | 4.89 (1.91) | 1.64 (1.63) | 1.59 (1.46) | 2.72 (2.15) | 0.79 (1.36) |
| **Baseline HAQ disability index** | 1.84 (0.57) | 1.51 (0.62) | 1.53 (0.61) | 1.73 (0.70) | 1.00 (0.73) |
| **Baseline ESR** | 47.86 (25.27) | 48.52 (27.74) | 46.39 (24.74) | 37.43 (26.59) | 24.85 (20.81) |
| **Baseline DAS28** | 6.44 (0.85) | 6.36 (0.95) | 6.54 (0.96) | 5.16 (1.26) | 4.26 ( 1.51) |

# Additional modelling framework

## Multi-arm studies and studies that do not include reference treatment

Here we generalize the model of Equation (5) described in Section 3.3 of the paper.

In case a study $j$ also includes a third treatment $Z$ we add a third arm in the expression for $m_{ij}$ in the first stage model. This study will then inform $\boldsymbol{\gamma}_{\boldsymbol{WA}}$ and $\boldsymbol{\gamma}_{\boldsymbol{ZA}}$, and also $\delta_{WA}$ and $\delta_{ZA}$. The random effects of the second stage model are instead

$$\left( \theta_{j,WA}^{(\delta)},\theta_{j,ZA}^{(\delta)} \right)\sim N\left( \left( \begin{matrix} \delta_{WA} \\ \delta_{ZA} \end{matrix} \right),\left( \begin{matrix} \tau^{2} & \tau^{2}/2 \\ \tau^{2}/2 & \tau^{2} \end{matrix} \right) \right)$$

Furthermore, for studies not including the reference treatment, we need to impose consistency in the second stage. For example, a two-arm study comparing $W$ vs. $Z$ will estimate $\boldsymbol{\gamma}_{\boldsymbol{WZ}}\boldsymbol{=}\boldsymbol{\gamma}_{\boldsymbol{WA}}\boldsymbol{-}\boldsymbol{\gamma}_{\boldsymbol{ZA}}$ and $\delta_{WZ}=\delta_{WA}-\delta_{ZA}$. Also note that this study will not estimate the intercept parameter $\alpha$. Thus, for such a study we will use the following likelihood in the second stage:

$$\left( {\hat{\boldsymbol{b}}}_{\boldsymbol{j}},{\hat{\boldsymbol{c}}}_{\boldsymbol{j},\boldsymbol{WZ}},\hat{d}_{j,WZ} \right)^{'} \sim N(\left( \boldsymbol{\beta},\boldsymbol{\gamma}_{\boldsymbol{WA}}-\boldsymbol{\gamma}_{\boldsymbol{ZA}}, \theta_{j,WZ}^{(\delta)} \right), {\hat{\boldsymbol{S}}}_{\boldsymbol{j}})$$

$$\theta_{j,WZ}^{(\delta)}\sim N\left( \delta_{WA}-\delta_{ZA}, \tau^{2} \right)$$

$$(\boldsymbol{\beta},\boldsymbol{\gamma}_{\boldsymbol{WA}}, {\boldsymbol{\gamma}_{\boldsymbol{ZA}},\delta}_{WA}, \delta_{ZA}, \tau^{2})\sim(vague prior distributions)$$

## Optimism-corrected performance

In what follows, we describe steps to obtain optimism-corrected measures of performance. First, for a given measure and for a given type of model $M$, we calculate optimism as follows. We draw a large number of bootstrap samples (e.g. N=200) and develop the model $M_{s}$ in each bootstrap sample $s$. We then calculate the **bootstrap performance** metric using the bootstrap sample $s$ and $M_{s}$. To find the **test performance**, we apply model $M_{s}$ to the original sample. **Optimism in sample** $\boldsymbol{s}$ **equals bootstrap performance minus test performance**. We repeat for all bootstrap samples, and we calculate **average optimism**. Lastly, we subtract average optimism from the performance of model $M$ in original sample (i.e. apparent performance). This is the optimism-corrected performance of model $M$ in our data.

## Reverting coefficients back to original scale after fitting the model

In order to use shrinkage models in each of the included studies, we first need to standardize the covariates in each study separately. Then we can fit the model in each study, but the meta-analysis of the second level becomes non-trivial, since in each study we use a different transformation. We show here how to address this issue.

Let us assume that we have a range of patient level covariates, so that for patient $i$ in study $j$ we have a vector $\boldsymbol{x}_{\boldsymbol{ij}}={(x}_{1ij}, x_{2ij}, \ldots)$. Let us also assume that in study $j$ we have average values $\bar{x_{1j}}, \bar{x_{2j}}\ldots$ and corresponding$sd_{1j}, sd_{2j}\ldots$ Finally let us assume that this patient received treatment $t$. For this patient, we rescale the covariates as $x_{1ij}^{*}=\frac{x_{1ij}-\bar{x_{1j}}}{sd_{1j}}$, $x_{2ij}^{*}=\frac{x_{2ij}-\bar{x_{2j}}}{sd_{2j}},\ldots$. The vector of rescaled covariates is thus

$$\boldsymbol{x}_{\boldsymbol{ij}}^{*}=\left( \frac{x_{1ij}}{sd_{1j}},\frac{x_{2ij}}{sd_{2j}},\ldots\right)-\left( \frac{\bar{x_{1j}}}{sd_{1j}},\frac{\bar{x_{2j}}}{sd_{2j}},\ldots\right)$$

If we set $\boldsymbol{m}_{\boldsymbol{j}}=\left( \frac{1}{sd_{1j}},\frac{1}{sd_{2j}},\ldots\right)$ and $\boldsymbol{n}_{\boldsymbol{j}}\boldsymbol{=}\left( -\frac{\bar{x_{1j}}}{sd_{1j}},-\frac{\bar{x_{2j}}}{sd_{2j}},\ldots\right)$, we can write that $\boldsymbol{x}^{\boldsymbol{*}}\boldsymbol{=}\boldsymbol{m}_{\boldsymbol{j}}⨀ \boldsymbol{x}_{\boldsymbol{ij}}\boldsymbol{+}\boldsymbol{n}_{\boldsymbol{j}}$, where the symbol $\boldsymbol{⨀}$ denotes element-wise multiplication,

Then we fit the following model: $y_{ij}=a^{*}+{\boldsymbol{\beta}^{\boldsymbol{*}}}^{\boldsymbol{T}}\boldsymbol{x}_{\boldsymbol{ij}}^{\boldsymbol{*}}\boldsymbol{+}{\boldsymbol{\gamma}_{\boldsymbol{tA}}^{\boldsymbol{*}}}^{\boldsymbol{T}}\boldsymbol{x}_{\boldsymbol{ij}}^{\boldsymbol{*}}+\delta_{tA}^{*}$, where $\boldsymbol{\gamma}_{\boldsymbol{AA}}^{\boldsymbol{*}}\boldsymbol{=}\delta_{AA}^{*}=0$. $A$ is the reference treatment in the network. We estimate the following vector $\boldsymbol{k}_{\boldsymbol{j}}^{\boldsymbol{*}}\boldsymbol{=(}a^{*}, \boldsymbol{\beta}^{\boldsymbol{*}}\boldsymbol{,}\boldsymbol{\gamma}_{\boldsymbol{BA}}^{\boldsymbol{*}}\boldsymbol{,}\boldsymbol{\gamma}_{\boldsymbol{CA}}^{\boldsymbol{*}}\boldsymbol{\ldots}\delta_{BA}^{*}, \delta_{CA}^{*}\ldots)'$ and the corresponding variance-covariance matrix $\boldsymbol{\Sigma}_{\boldsymbol{j}}^{\boldsymbol{*}}\boldsymbol{=}var\boldsymbol{(}\boldsymbol{k}_{\boldsymbol{j}}^{\boldsymbol{*}}\boldsymbol{)}$. We now note that the model can be rewritten as follows:

$$y_{ij}=a^{*}+{\boldsymbol{\beta}^{\boldsymbol{*}}}^{\boldsymbol{T}}\left( \boldsymbol{m}_{\boldsymbol{j}} \boldsymbol{⨀}\boldsymbol{x}_{\boldsymbol{ij}}\boldsymbol{+}\boldsymbol{n}_{\boldsymbol{j}} \right)+{\boldsymbol{\gamma}_{\boldsymbol{tA}}^{\boldsymbol{*}}}^{\boldsymbol{T}}\left( \boldsymbol{m}_{\boldsymbol{j}} \boldsymbol{⨀}\boldsymbol{x}_{\boldsymbol{ij}}\boldsymbol{+}\boldsymbol{n}_{\boldsymbol{j}} \right)+\delta_{tA}^{*}=$$

$$=\left( a^{*}+{\boldsymbol{\beta}^{\boldsymbol{*}}}^{\boldsymbol{T}}\boldsymbol{n}_{\boldsymbol{j}} \right)\boldsymbol{+}{\boldsymbol{\beta}^{\boldsymbol{*}}}^{\boldsymbol{T}}\boldsymbol{m}_{\boldsymbol{j}} \boldsymbol{⨀}\boldsymbol{x}_{\boldsymbol{ij}}\boldsymbol{+}{\boldsymbol{\gamma}_{\boldsymbol{tA}}^{\boldsymbol{*}}}^{\boldsymbol{T}}\boldsymbol{m}_{\boldsymbol{j}} \boldsymbol{⨀}\boldsymbol{x}_{\boldsymbol{ij}}\boldsymbol{+(}\delta_{tA}^{*}+{\boldsymbol{\gamma}_{\boldsymbol{tA}}^{\boldsymbol{*}}}^{\boldsymbol{T}}\boldsymbol{n}_{\boldsymbol{j}}\boldsymbol{)}$$

$$\boldsymbol{=}\left( a^{*}-\beta_{1}^{*}\frac{\bar{x_{1j}}}{sd_{1j}}-\beta_{2}^{*}\frac{\bar{x_{2j}}}{sd_{2j}}\ldots\right)\boldsymbol{+}\left( \beta_{1}^{*}\frac{1}{sd_{1j}}x_{1ij}\boldsymbol{+}\beta_{2}^{*}\frac{1}{sd_{2j}}x_{2ij}\boldsymbol{+\ldots} \right)\boldsymbol{+}$$

$$\left( \gamma_{1tA}^{*}\frac{1}{sd_{1j}}x_{1ij}\boldsymbol{+}\gamma_{2tA}^{*}\frac{1}{sd_{2j}}x_{2ij}\boldsymbol{+\ldots} \right)\boldsymbol{+}\left( \delta_{tA}^{*}-\gamma_{1tA}^{*}\frac{\bar{x_{1j}}}{sd_{1j}}-\gamma_{2tA}^{*}\frac{\bar{x_{2j}}}{sd_{2j}}-\ldots\right)$$

This is equivalent to a model with the covariates in the original scale. In other words, the above expression is the same as

$$y_{ij}=a\boldsymbol{+}\boldsymbol{\beta}^{\boldsymbol{T}}\boldsymbol{x}_{\boldsymbol{ij}}\boldsymbol{+}\boldsymbol{\gamma}_{\boldsymbol{tA}}^{\boldsymbol{T}} \boldsymbol{x}_{\boldsymbol{ij}}\boldsymbol{+}\delta_{tA}$$

if we set $a=a^{*}+{\boldsymbol{\beta}^{\boldsymbol{*}}}^{\boldsymbol{T}}\boldsymbol{n}_{\boldsymbol{j}}$, $\boldsymbol{\beta}\boldsymbol{=}\boldsymbol{\beta}^{\boldsymbol{*}}\boldsymbol{⨀}\boldsymbol{m}_{\boldsymbol{j}}$, $\boldsymbol{\gamma}_{\boldsymbol{tA}}\boldsymbol{=}\boldsymbol{\gamma}_{\boldsymbol{tA}}^{\boldsymbol{*}}\boldsymbol{⨀ m}_{\boldsymbol{j}}$ **,**$\delta_{tA}=\delta_{tA}^{*}+{\boldsymbol{\gamma}_{\boldsymbol{tA}}^{\boldsymbol{*}}}^{\boldsymbol{T}}\boldsymbol{n}_{\boldsymbol{j}}\boldsymbol{.}$To write it more compactly, the parameters $\boldsymbol{k}_{\boldsymbol{j}}=\left( \alpha,\boldsymbol{\beta,}\boldsymbol{\gamma}_{\boldsymbol{BA}}\boldsymbol{,}\boldsymbol{\gamma}_{\boldsymbol{CA}},\ldots\delta_{BA}, \delta_{CA}\ldots\right)$are just linear combinations of the estimated parameters in $\boldsymbol{k}_{\boldsymbol{j}}^{\boldsymbol{*}}$ i.e. $\boldsymbol{k}_{\boldsymbol{j}}\boldsymbol{=}\boldsymbol{N}_{\boldsymbol{j}}^{\boldsymbol{*}}\boldsymbol{k}_{\boldsymbol{j}}^{\boldsymbol{*}}$, where $\boldsymbol{N}_{\boldsymbol{j}}^{\boldsymbol{*}}$ is a transformation matrix which can be written as a function of $\boldsymbol{m}_{\boldsymbol{j}}$ and $\boldsymbol{n}_{\boldsymbol{j}}$**.** As an example, the first row of $\boldsymbol{N}_{\boldsymbol{j}}^{\boldsymbol{*}}$ is $\left( 1, \boldsymbol{n}_{\boldsymbol{j}},\ldots,0,0,0\ldots\right)=\left( 1, -\frac{\bar{x_{1j}}}{sd_{1j}},-\frac{\bar{x_{2j}}}{sd_{2j}}\ldots,0,0,0\ldots\right)$. The variance-covariance matrix of $\boldsymbol{k}_{\boldsymbol{j}}$ is $var\left( \boldsymbol{k}_{\boldsymbol{j}} \right)=\boldsymbol{\Sigma}=\boldsymbol{N}_{\boldsymbol{j}}^{*}var\left( \boldsymbol{k}_{\boldsymbol{j}}^{*} \right){\boldsymbol{N}_{\boldsymbol{j}}^{*}}^{\boldsymbol{T}}=\boldsymbol{N}_{\boldsymbol{j}}^{*}\boldsymbol{\Sigma}_{\boldsymbol{j}}^{*}{\boldsymbol{N}_{\boldsymbol{j}}^{*}}^{\boldsymbol{T}}$

In summary, we follow these steps:

- Transform the covariates $\boldsymbol{x\to}\boldsymbol{x}^{\boldsymbol{*}}$ in each study according to the study-specific mean and standard deviation.
- Perform the analysis with shrinkage on the $\boldsymbol{x}^{\boldsymbol{*}}$. Estimate the corresponding coefficients $\boldsymbol{k}_{\boldsymbol{j}}^{\boldsymbol{*}}$ and their variance-covariance matrix $\boldsymbol{\Sigma}_{\boldsymbol{j}}^{\boldsymbol{*}}$.
- Back-transform the estimated means and variance covariance matrix to estimate $\boldsymbol{k}_{\boldsymbol{j}}$ and $\boldsymbol{\Sigma}$ using the formulas above, to build a model that works on the natural scale, i.e. $\boldsymbol{x}$.

We now have the same model in each study (i.e. with covariates measured on the same scale), and we can meta-analyse the $\boldsymbol{k}_{\boldsymbol{j}}$.

# Detailed results from the analysis of the rheumatoid arthritis example

**Table 4**: First stage linear mixed effects model results for Approach II(a) prior to reverting to unstandardized coefficients. Numbers in parentheses are standard deviations of the posterior distributions. Abbreviations: RTX: Rituximab; TCZ: Tocilizumab; BMI: Body mass index; DMARD: Disease-modifying anti-rheumatic Drug; anti-TNF: anti-tumor necrosis factor; HAQ: health assessment questionnaire; ESR: erythrocyte sedimentation rate; DAS28: Disease Activity Score 28.

| **Parameter** | | **REFLEX** | **TOWARD** | **TOWARD2** | **BSRBR-RA** | **SCQM** |
| --- | --- | --- | --- | --- | --- | --- |
| Study intercept | | 5.74 (0.12) | 5.38 (0.07) | 4.96 (0.09) | 4.73 (0.09) | 3.52 (0.05) |
| Treatment effect of RTX | | -1.14 (0.15) | - |  | -0.65 (0.11) | -0.22 (0.24) |
| Treatment effect of TCZ | | - | -1.91 (0.09) | -1.70 (0.12) | -1.93 (0.14) | -1.57 (0.39) |
| Residual standard deviation ($\sigma_{\epsilon}$) | | 1.30 (0.05) | 1.30 (0.03) | 1.29 (0.04) | 1.21 (0.03) | 1.21 (0.03) |
| **Main**  **effects** | Female | 0.01 (0.11) | 0.06 (0.08) | 0.15 (0.10) | 0.15 (0.07) | 0.12 (0.05) |
|  | Age | -0.04 (0.13) | 0.03 (0.08) | -0.12 (0.09) | -0.04 (0.07) | 0.01 (0.05) |
|  | disease duration | -0.08 (0.15) | -0.05 (0.08) | 0.02 (0.11) | -0.06 (0.07) | 0.12 (0.05) |
|  | BMI | 0.06 (0.12) | 0.08 (0.07) | 0.06 (0.09) | 0.03 (0.06) | 0.05 (0.06) |
|  | baseline rheumatoid factor | 0.05 (0.13) | 0.03 (0.07) | 0.19 (0.09) | 0.01 (0.07) | 0.17 (0.05) |
|  | Number of previous DMARDs/anti-TNFs | 0.16 (0.15) | 0.04 (0.08) | 0.02 (0.11) | 0.15 (0.07) | 0.10 (0.08) |
|  | baseline HAQ | -0.06 (0.14) | 0.12 (0.08) | 0.08 (0.12) | 0.07 (0.10) | 0.12 (0.06) |
|  | baseline ESR | -0.08 (0.14) | 0.18 (0.09) | 0.18 (0.11) | 0.04 (0.07) | 0.07 (0.06) |
|  | baseline DAS28 | 0.69 (0.16) | 0.48 (0.09) | 0.45 (0.13) | 0.70 (0.07) | 0.59 (0.06) |
| **Effect modifiers with RTX**  **(treatment-covariate interaction)** | Female | 0.03 (0.14) | - |  | 0.00 (0.09) | 0.08 (0.14) |
|  | Age | 0.06 (0.16) | - |  | 0.04 (0.09) | 0.10 (0.14) |
|  | disease duration | 0.04 (0.17) | - |  | 0.09 (0.10) | -0.31 (0.14) |
|  | BMI | 0.21 (0.15) | - |  | -0.02 (0.07) | 0.19 (0.13) |
|  | baseline rheumatoid factor | -0.24 (0.15) | - |  | -0.08 (0.09) | -0.20 (0.16) |
|  | Number of previous DMARDs/anti-TNFs | -0.14 (0.17) | - |  | -0.22 (0.10) | -0.05 (0.15) |
|  | baseline HAQ | 0.10 (0.16) | - |  | 0.12 (0.13) | -0.02 (0.18) |
|  | baseline ESR | -0.04 (0.17) | - |  | 0.05 (0.10) | -0.09 (0.15) |
|  | baseline DAS28 | 0.09 (0.18) | - |  | -0.20 (0.11) | 0.12 (0.19) |
| **Effect modifiers with TCZ**  **(treatment-covariate interaction)** | female | - | -0.01 (0.10) | -0.08 (0.12) | -0.19 (0.13) | -0.08 (0.21) |
|  | age | - | 0.05 (0.09) | 0.19 (0.12) | 0.01 (0.12) | 0.13 (0.32) |
|  | disease duration | - | -0.02 (0.10) | -0.04 (0.14) | 0.02 (0.14) | -0.17 (0.22) |
|  | BMI | - | -0.05 (0.09) | -0.14 (0.13) | 0.24 (0.22) | 0.10 (0.21) |
|  | baseline rheumatoid factor | - | -0.18 (0.09) | -0.13 (0.12) | -0.08 (0.13) | -0.15 (0.18) |
|  | Number of previous DMARDs/anti-TNFs | - | 0.14 (0.10) | -0.14 (0.14) | -0.10 (0.15) | 0.16 (0.23) |
|  | baseline HAQ | - | -0.03 (0.10) | 0.04 (0.15) | 0.15 (0.17) | -0.15 (0.28) |
|  | baseline ESR | - | -0.19 (0.11) | -0.04 (0.14) | 0.00 (0.15) | 0.02 (0.23) |
|  | baseline DAS28 | - | 0.10 (0.11) | -0.01 (0.16) | -0.53 (0.15) | -0.10 (0.37) |

**Table 5**: First stage linear mixed effects model results for Approach II(a). Numbers in parentheses are standard deviations of the posterior distributions. Abbreviations: RTX: Rituximab; TCZ: Tocilizumab; BMI: Body mass index; DMARD: Disease-modifying anti-rheumatic Drug; anti-TNF: anti-tumor necrosis factor; HAQ: health assessment questionnaire; ESR: erythrocyte sedimentation rate; DAS28: Disease Activity Score 28.

| **Parameter** | | **REFLEX** | **TOWARD** | **TOWARD2** | **BSRBR-RA** | **SCQM** |
| --- | --- | --- | --- | --- | --- | --- |
| Study intercept | | 0.35 (1.25) | 0.85 (0.74) | 0.81 (0.88) | 1.27 (0.45) | 0.44 (0.36) |
| Treatment effect of RTX | | -2.46 (1.50) | - | - | -0.08 (0.67) | -1.22 (1.10) |
| Treatment effect of TCZ | | - | -1.98 (0.86) | -1.21 (1.15) | -0.28 (0.94) | -1.49 (1.93) |
| Residual standard deviation ($\sigma_{\epsilon}$) | | 1.30 (0.05) | 1.30 (0.03) | 1.29 (0.04) | 1.21 (0.03) | 1.21 (0.03) |
| **Main**  **effects** | Female | 0.03 (0.29) | 0.15 (0.21) | 0.39 (0.25) | 0.35 (0.16) | 0.27 (0.10) |
|  | Age | -0.00 (0.01) | 0.00 (0.01) | -0.01 (0.01) | -0.00 (0.01) | 0.00 (0.00) |
|  | disease duration | -0.01 (0.02) | -0.01 (0.01) | 0.00 (0.01) | -0.01 (0.01) | 0.01 (0.01) |
|  | BMI | 0.01 (0.02) | 0.01 (0.01) | 0.01 (0.01) | 0.00 (0.01) | 0.02 (0.01) |
|  | baseline rheumatoid factor | 0.13 (0.31) | 0.06 (0.17) | 0.50 (0.24) | 0.02 (0.14) | 0.44 (0.11) |
|  | Number of previous DMARDs/anti-TNFs | 0.08 (0.08) | 0.03 (0.05) | 0.01 (0.08) | 0.06 (0.03) | 0.08 (0.06) |
|  | baseline HAQ | -0.10 (0.24) | 0.20 (0.13) | 0.13 (0.19) | 0.29 (0.15) | 0.18 (0.08) |
|  | baseline ESR | -0.00 (0.01) | 0.01 (0.00) | 0.01 (0.00) | 0.00 (0.00) | 0.00 (0.00) |
|  | baseline DAS28 | 0.81 (0.19) | 0.51 (0.10) | 0.47 (0.14) | 0.53 (0.06) | 0.39 (0.04) |
| **Effect modifiers with RTX**  **(treatment-covariate interaction)** | Female | 0.07 (0.36) | - | - | 0.00 (0.22) | 0.17 (0.31) |
|  | Age | 0.00 (0.01) | - | - | 0.00 (0.01) | 0.01 (0.01) |
|  | disease duration | 0.01 (0.02) | - | - | 0.01 (0.01) | -0.04 (0.02) |
|  | BMI | 0.03 (0.02) | - | - | -0.00 (0.01) | 0.03 (0.03) |
|  | baseline rheumatoid factor | -0.57 (0.37) | - | - | -0.17 (0.19) | -0.49 (0.37) |
|  | Number of previous DMARDs/anti-TNFs | -0.07 (0.09) | - | - | -0.10 (0.05) | -0.04 (0.11) |
|  | baseline HAQ | 0.17 (0.29) | - | - | 0.03 (0.19) | -0.02 (0.22) |
|  | baseline ESR | -0.00 (0.01) | - | - | 0.00 (0.00) | -0.00 (0.01) |
|  | baseline DAS28 | 0.11 (0.22) | - | - | -0.13 (0.08) | 0.08 (0.13) |
| **Effect modifiers with TCZ**  **(treatment-covariate interaction)** | female | - | -0.04 (0.25) | -0.21 (0.32) | -0.43 (0.30) | -0.16 (0.47) |
|  | age | - | 0.00 (0.01) | 0.02 (0.01) | 0.00 (0.01) | 0.01 (0.02) |
|  | disease duration | - | -0.00 (0.01) | -0.00 (0.02) | 0.01 (0.01) | -0.02 (0.03) |
|  | BMI | - | -0.01 (0.01) | -0.02 (0.02) | 0.02 (0.02) | 0.01 (0.04) |
|  | baseline rheumatoid factor | - | -0.42 (0.21) | -0.35 (0.31) | -0.25 (0.29) | -0.36 (0.43) |
|  | Number of previous DMARDs/anti-TNFs | - | 0.08 (0.06) | -0.09 (0.10) | -0.04 (0.07) | 0.11 (0.17) |
|  | baseline HAQ | - | -0.04 (0.16) | 0.06 (0.24) | 0.08 (0.22) | -0.02 (0.40) |
|  | baseline ESR | - | -0.01 (0.00) | -0.00 (0.01) | 0.00 (0.01) | 0.00 (0.01) |
|  | baseline DAS28 | - | 0.11 (0.12) | -0.01 (0.17) | -0.39 (0.11) | -0.13 (0.24) |

**Table 6**: First stage linear mixed effects model results incorporating shrinkage on effect modifiers (i.e. Bayesian LASSO) for Approach II(b). Numbers in parentheses are standard deviations of the posterior distributions. Abbreviations: RTX: Rituximab; TCZ: Tocilizumab; BMI: Body mass index; DMARD: Disease-modifying anti-rheumatic Drug; anti-TNF: anti-tumor necrosis factor; HAQ: health assessment questionnaire; ESR: erythrocyte sedimentation rate; DAS28: Disease Activity Score 28.

| **Parameter** | | **REFLEX** | **TOWARD** | **TOWARD2** | **BSRBR-RA** | **SCQM** |
| --- | --- | --- | --- | --- | --- | --- |
| Study intercept | | -0.35 (0.85) | 0.66 (0.51) | 0.98 (0.63) | 1.52 (0.37) | 0.33 (0.35) |
| Treatment effect of RTX | | -1.40 (0.74) | - | - | -0.47 (0.46) | -0.48 (0.55) |
| Treatment effect of TCZ | | - | -1.77 (0.47) | -1.58 (0.53) | -0.84 (0.71) | -1.26 (0.65) |
| Residual standard deviation ($\sigma_{\epsilon}$) | | 1.30 (0.05) | 1.30 (0.05) | 1.28 (0.04) | 1.21 (0.03) | 1.20 (0.03) |
| **Main**  **effects** | Female | 0.02 (0.20) | 0.13 (0.14) | 0.28 (0.18) | 0.29 (0.12) | 0.28 (0.10) |
|  | Age | -0.00 (0.01) | 0.00 (0.00) | -0.00 (0.01) | -0.00 (0.00) | 0.00 (0.00) |
|  | disease duration | -0.01 (0.01) | -0.01 (0.01) | 0.00 (0.01) | -0.00 (0.01) | 0.01 (0.01) |
|  | BMI | 0.02 (0.01) | 0.01 (0.01) | -0.00 (0.01) | 0.00 (0.00) | 0.02 (0.01) |
|  | baseline rheumatoid factor | -0.20 (0.22) | -0.10 (0.15) | 0.34 (0.18) | -0.10 (0.11) | 0.38 (0.10) |
|  | Number of previous DMARDs/anti-TNFs | 0.04 (0.05) | 0.07 (0.04) | -0.04 (0.05) | 0.04 (0.03) | 0.07 (0.05) |
|  | baseline HAQ | 0.02 (0.16) | 0.17 (0.09) | 0.16 (0.13) | 0.32 (0.11) | 0.15 (0.07) |
|  | baseline ESR | -0.00 (0.00) | 0.00 (0.00) | 0.01 (0.00) | 0.00 (0.00) | 0.00 (0.00) |
|  | baseline DAS28 | 0.87 (0.11) | 0.59 (0.07) | 0.47 (0.09) | 0.46 (0.05) | 0.40 (0.04) |
| **Effect modifiers with RTX**  **(treatment-covariate interaction)** | Female | 0.03 (0.16) | - | - | 0.03 (0.13) | 0.04 (0.14) |
|  | Age | 0.00 (0.01) | - | - | 0.00 (0.00) | 0.00 (0.00) |
|  | disease duration | 0.00 (0.01) | - | - | 0.00 (0.01) | -0.01 (0.01) |
|  | BMI | 0.01 (0.01) | - | - | -0.00 (0.00) | 0.01 (0.01) |
|  | baseline rheumatoid factor | -0.11 (0.21) | - | - | -0.07 (0.13) | -0.09 (0.19) |
|  | Number of previous DMARDs/anti-TNFs | -0.01 (0.04) | - | - | -0.04 (0.04) | -0.01 (0.05) |
|  | baseline HAQ | 0.03 (0.12) | - | - | -0.02 (0.10) | -0.00 (0.09) |
|  | baseline ESR | -0.00 (0.00) | - | - | 0.00 (0.00) | -0.00 (0.00) |
|  | baseline DAS28 | 0.02 (0.08) | - | - | -0.04 (0.06) | 0.00(0.04) |
| **Effect modifiers with TCZ**  **(treatment-covariate interaction)** | female | - | -0.01 (0.12) | -0.05 (0.15) | -0.17 (0.20) | -0.04 (0.16) |
|  | age | - | 0.00 (0.00) | 0.00 (0.01) | 0.00 (0.01) | 0.00 (0.01) |
|  | disease duration | - | 0.00 (0.01) | -0.00 (0.01) | -0.00 (0.01) | -0.00 (0.01) |
|  | BMI | - | -0.00 (0.01) | -0.00 (0.01) | 0.01 (0.01) | 0.00 (0.01) |
|  | baseline rheumatoid factor | - | -0.16 (0.18) | -0.08 (0.15) | -0.07 (0.16) | -0.08 (0.18) |
|  | Number of previous DMARDs/anti-TNFs | - | 0.02 (0.04) | -0.02 (0.04) | -0.00 (0.03) | 0.01 (0.05) |
|  | baseline HAQ | - | -0.01 (0.08) | -0.00 (0.09) | 0.00 (0.10) | -0.01 (0.10) |
|  | baseline ESR | - | -0.00 (0.00) | -0.00 (0.00) | -0.00 (0.00) | 0.00 (0.00) |
|  | baseline DAS28 | - | 0.01 (0.05) | -0.00 (0.06) | -0.19 (0.11) | -0.01 (0.05) |

**Table 7**: First stage linear mixed effects model results for Approach IV. Numbers in parentheses are standard deviations of the posterior distributions. Abbreviations: RTX: Rituximab; TCZ: Tocilizumab; BMI: Body mass index; DMARD: Disease-modifying anti-rheumatic Drug; anti-TNF: anti-tumor necrosis factor; HAQ: health assessment questionnaire; ESR: erythrocyte sedimentation rate; DAS28: Disease Activity Score 28.

| **Parameter** | | **REFLEX** | **TOWARD** | **TOWARD2**^1^ | **BSRBR-RA** | **SCQM** |
| --- | --- | --- | --- | --- | --- | --- |
| Study intercept | | -0.52 (0.70) | 0.74 (0.39) | 0.98 (0.63) | 1.72 (0.30) | 0.25 (0.34) |
| Treatment effect of RTX | | -1.12 (0.15) | - | - | -0.68 (0.09) | -0.43 (0.18) |
| Treatment effect of TCZ | | - | -1.91 (0.09) | -1.58 (0.53) | -1.99 (0.12) | -1.23 (0.23) |
| Residual standard deviation ($\sigma_{\epsilon}$) | | 1.30 (0.05) | 1.30 (0.03) | 1.28 (0.04) | 1.21 (0.03) | 1.20 (0.03) |
| **Main**  **effects** | Female | 0.04 (0.17) | 0.12 (0.11) | 0.28 (0.18) | 0.28 (0.10) | 0.29 (0.09) |
|  | Age | -0.00 (0.01) | 0.00 (0.00) | -0.00 (0.01) | -0.00 (0.00) | 0.00 (0.00) |
|  | disease duration | -0.01 (0.01) | -0.01 (0.01) | 0.00 (0.01) | -0.00 (0.00) | 0.00 (0.01) |
|  | BMI | 0.03 (0.01) | 0.01 (0.01) | -0.00 (0.01) | 0.00 (0.00) | 0.03 (0.01) |
|  | baseline rheumatoid factor | -0.27 (0.17) | -0.20 (0.10) | 0.34 (0.18) | -0.12 (0.09) | 0.38 (0.10) |
|  | Number of previous DMARDs/anti-TNFs | 0.03 (0.04) | 0.08 (0.03) | -0.04 (0.05) | 0.02 (0.02) | 0.07 (0.05) |
|  | baseline HAQ | 0.05 (0.13) | 0.16 (0.07) | 0.16 (0.13) | 0.31 (0.08) | 0.15 (0.07) |
|  | baseline ESR | -0.00 (0.00) | 0.00 (0.00) | 0.01 (0.00) | 0.00 (0.00) | 0.00 (0.00) |
|  | baseline DAS28 | 0.88 (0.09) | 0.60 (0.05) | 0.47 (0.09) | 0.42 (0.04) | 0.40 (0.04) |

*^1^ Due to the terms in the data-sharing agreement, the data from the indicated RCT were no longer available at the time Approach IV was added in the manuscript (during the revision of the paper). Thus, for this particular RCT and especially for Approach IV, we used estimates from the first stage linear mixed effects model using Bayesian LASSO (i.e. Approach IIb). Results are expected to be very similar, given that Approach IIb gave almost zero estimates for all effect modifiers in this study.*

**Table 8**: Second stage model results meta-analyzing all the studies for different approaches. Numbers in parentheses are standard deviations of the posterior distributions. Abbreviations: RTX: Rituximab; TCZ: Tocilizumab; BMI: Body mass index; DMARD: Disease-modifying anti-rheumatic Drug; anti-TNF: anti-tumor necrosis factor; HAQ: health assessment questionnaire; ESR: erythrocyte sedimentation rate; DAS28: Disease Activity Score 28.

| **Parameter** | | **Approach IIa** | **Approach IIb** | **Approach IIc** | **Approach IIIa**  **(w=0.25)** | **Approach IIIa**  **(w=0.5)** | **Approach IIIb**  **(w=0.25)** | **Approach IIIb**  **(w=0.5)** | **Approach IV** |
| --- | --- | --- | --- | --- | --- | --- | --- | --- | --- |
| Intercept ($a$ parameter of the prediction model) | | 0.61 (0.20) | 0.76 (0.16) | N/A^1^ | 0.89 (0.20) | 0.91 (0.20) | N/A^1^ | NA^1^ | 0.81 (0.15) |
| Treatment effect of RTX | | 0.10 (0.41) | -0.32 (0.26) | -0.32 (0.26) | -0.43 (0.44) | -0.34 (0.35) | -0.43 (0.44) | -0.34 (0.35) | -0.68 (0.06) |
| Treatment effect of TCZ | | -0.97 (0.38) | -1.42 (0.24) | -1.42 (0.24) | -1.56 (0.32) | -1.48 (0.30) | -1.56 (0.32) | -1.48 (0.30) | -1.62 (0.05) |
| **Main**  **Effects**  ($\boldsymbol{\beta}$ parameters of the prediction model) | female | 0.27 (0.07) | 0.24 (0.06) | N/A^1^ | 0.27 (0.07) | 0.27 (0.07) | N/A^1^ | NA^1^ | 0.22 (0.05) |
|  | age | -0.00 (0.00) | 0.00 (0.00) |  | -0.00 (0.00) | 0.00 (0.00) |  |  | 0.00 (0.00) |
|  | disease duration | 0.00 (0.00) | 0.00 (0.00) |  | 0.00 (0.00) | 0.00 (0.00) |  |  | 0.00 (0.00) |
|  | BMI | 0.01 (0.00) | 0.01 (0.00) |  | 0.01 (0.00) | 0.01 (0.00) |  |  | 0.01 (0.00) |
|  | baseline rheumatoid factor | 0.21 (0.07) | 0.03 (0.06) |  | 0.09 (0.07) | 0.09 (0.07) |  |  | -0.02 (0.05) |
|  | Number of previous DMARDs/anti-TNFs | 0.12 (0.02) | 0.09 (0.01) |  | 0.10 (0.02) | 0.11 (0.02) |  |  | 0.07 (0.01) |
|  | baseline HAQ | 0.19 (0.05) | 0.21 (0.04) |  | 0.29 (0.05) | 0.28 (0.05) |  |  | 0.21 (0.04) |
|  | baseline ESR | 0.00 (0.00) | 0.00 (0.00) |  | 0.00 (0.00) | 0.00 (0.00) |  |  | 0.00 (0.00) |
|  | baseline DAS28 | 0.50 (0.03) | 0.50 (0.02) |  | 0.43 (0.03) | 0.43 (0.03) |  |  | 0.50 (0.02) |
| **Effect modifiers with RTX**  **(treatment-covariate interaction,** $\boldsymbol{\gamma}$ parameters of the prediction model) | female | -0.01 (0.13) | 0.01 (0.08) | 0.01 (0.08) | 0.04 (0.12) | 0.04 (0.10) | 0.04 (0.12) | 0.04 (0.10) |  |
|  | age | 0.00 (0.00) | 0.00 (0.00) | 0.00 (0.00) | 0.00 (0.00) | 0.00 (0.00) | 0.00 (0.00) | 0.00 (0.00) |  |
|  | disease duration | -0.01 (0.01) | -0.00 (0.00) | -0.00 (0.00) | -0.00 (0.01) | -0.00 (0.01) | -0.00 (0.01) | -0.00 (0.01) |  |
|  | BMI | -0.01 (0.01) | -0.00 (0.00) | -0.00 (0.00) | -0.00 (0.01) | -0.00 (0.01) | -0.00 (0.01) | -0.00 (0.01) |  |
|  | baseline rheumatoid factor | -0.48 (0.12) | -0.14 (0.08) | -0.14 (0.08) | -0.17 (0.13) | -0.15 (0.11) | -0.17 (0.13) | -0.15 (0.11) |  |
|  | Number of previous DMARDs/anti-TNFs | -0.13 (0.03) | -0.06 (0.02) | -0.06 (0.02) | -0.06 (0.03) | -0.06 (0.03) | -0.06 (0.03) | -0.06 (0.03) |  |
|  | baseline HAQ | 0.09 (0.09) | 0.01 (0.05) | 0.01 (0.05) | 0.03 (0.08) | 0.02 (0.07) | 0.03 (0.08) | 0.02 (0.07) |  |
|  | baseline ESR | -0.00 (0.00) | -0.00 (0.00) | -0.00 (0.00) | -0.00 (0.00) | -0.00 (0.00) | -0.00 (0.00) | -0.00 (0.00) |  |
|  | baseline DAS28 | -0.01 (0.05) | -0.01 (0.03) | -0.01 (0.03) | -0.01 (0.05) | -0.01 (0.04) | -0.01 (0.05) | -0.01 (0.04) |  |
| **Effect modifiers with TCZ**  **(treatment-covariate interaction,** $\boldsymbol{\gamma}$ parameters of the prediction model**)** | female | -0.15 (0.12) | -0.05 (0.07) | -0.05 (0.07) | -0.03 (0.09) | -0.03 (0.08) | -0.03 (0.09) | -0.03 (0.08) |  |
|  | age | 0.01 (0.00) | 0.00 (0.00) | 0.00 (0.00) | 0.00 (0.00) | 0.00 (0.00) | 0.00 (0.00) | 0.00 (0.00) |  |
|  | disease duration | -0.01 (0.01) | -0.00 (0.00) | -0.00 (0.00) | -0.00 (0.00) | -0.00 (0.00) | -0.00 (0.00) | -0.00 (0.00) |  |
|  | BMI | -0.01 (0.01) | -0.00 (0.00) | -0.00 (0.00) | -0.00 (0.01) | -0.00 (0.01) | -0.00 (0.01) | -0.00 (0.01) |  |
|  | baseline rheumatoid factor | -0.42 (0.11) | -0.10 (0.07) | -0.10 (0.07) | -0.10 (0.10) | -0.10 (0.09) | -0.10 (0.10) | -0.10 (0.09) |  |
|  | Number of previous DMARDs/anti-TNFs | -0.06 (0.03) | -0.02 (0.02) | -0.02 (0.02) | -0.00 (0.02) | -0.00 (0.02) | -0.00 (0.02) | -0.00 (0.02) |  |
|  | baseline HAQ | -0.02 (0.08) | -0.03 (0.04) | -0.03 (0.04) | -0.00 (0.05) | -0.01 (0.05) | -0.00 (0.05) | -0.01 (0.05) |  |
|  | baseline ESR | -0.00 (0.00) | -0.00 (0.00) | -0.00 (0.00) | -0.00 (0.00) | -0.00 (0.00) | -0.00 (0.00) | -0.00 (0.00) |  |
|  | baseline DAS28 | -0.02 (0.05) | -0.01 (0.03) | -0.01 (0.03) | -0.01 (0.04) | -0.02 (0.03) | -0.01 (0.04) | -0.02 (0.03) |  |

*^1^* For the case of α and β, parameters are defined according to where the model will be used.

**Table 9**: Internal validation results corrected for optimism of the performance metric for different approaches. Abbreviations: MSE: mean squared error; DMARDs: disease-modifying anti-rheumatic drugs; RTX: Rituximab; TCZ: Tocilizumab.

| **Dataset** | **Treatment arms** | **Performance metric** | **Approach I** | **Approach IIa** | **Approach IIb** | **Approach IIc** | **Approach IIIa**  **w=0.25** | **Approach IIIa**  **w = 0.5** | **Approach IIIb**  **w=0.25** | **Approach IIIb**  **w = 0.5** | **Approach IV** |
| --- | --- | --- | --- | --- | --- | --- | --- | --- | --- | --- | --- |
| SCQM | All arms | R-squared | 0.26 | 0.23 | 0.21 | 0.26 | 0.24 | 0.24 | 0.26 | 0.26 | 0.22 |
|  |  | MSE | 1.50 | 1.56 | 1.60 | 1.50 | 1.55 | 1.55 | 1.51 | 1.51 | 1.61 |
|  |  | Bias | 0.10 | 0.22 | 0.25 | 0.05 | 0.18 | 0.19 | 0.04 | 0.04 | 0.26 |
|  | DMARDs | MSE | 1.60 | 1.69 | 1.74 | 1.60 | 1.67 | 1.66 | 1.60 | 1.60 | 1.74 |
|  |  | Bias | 0.12 | 0.27 | 0.32 | 0.12 | 0.25 | 0.24 | 0.12 | 0.12 | 0.34 |
|  | RTX + DMARDs | MSE | 1.03 | 1.05 | 1.02 | 0.98 | 1.03 | 1.04 | 0.99 | 0.99 | 1.04 |
|  |  | Bias | 0.12 | 0.13 | 0.10 | -0.09 | 0.05 | 0.11 | -0.12 | -0.12 | 0.05 |
|  | TCZ + DMARDs | MSE | 1.37 | 1.05 | 1.14 | 1.47 | 1.28 | 1.27 | 1.58 | 1.58 | 1.14 |
|  |  | Bias | -0.17 | -0.26 | -0.37 | -0.58 | -0.49 | -0.46 | -0.67 | -0.67 | -0.36 |
| BSRBR-RA | All arms | R-squared | 0.36 | 0.36 | 0.36 | 0.34 | 0.37 | 0.37 | 0.35 | 0.35 | 0.36 |
|  |  | MSE | 1.47 | 1.47 | 1.46 | 1.50 | 1.45 | 1.45 | 1.48 | 1.48 | 1.46 |
|  |  | Bias | 0.14 | 0.12 | 0.08 | 0.20 | 0.05 | 0.06 | 0.18 | 0.18 | 0.05 |
|  | DMARDs | MSE | 1.51 | 1.45 | 1.41 | 1.51 | 1.46 | 1.46 | 1.50 | 1.50 | 1.39 |
|  |  | Bias | 0.16 | 0.08 | 0.06 | 1.63 | 0.10 | 0.09 | 0.16 | 0.16 | 0.02 |
|  | RTX + DMARDs | MSE | 1.34 | 1.33 | 1.34 | 1.32 | 1.33 | 1.33 | 1.31 | 1.31 | 1.36 |
|  |  | Bias | 0.12 | 0.10 | 0.05 | 0.15 | -0.01 | 0.02 | 0.15 | 0.15 | 0.00 |
|  | TCZ + DMARDs | MSE | 1.80 | 2.00 | 2.00 | 2.11 | 1.84 | 1.83 | 2.00 | 2.01 | 2.03 |
|  |  | Bias | 0.16 | 0.29 | 0.24 | 0.44 | 0.11 | 0.11 | 0.36 | 0.36 | 0.28 |

**Figure 1**: Calibration plot from internal validation, for the British registry as the external dataset. Black line is line of perfect calibration. Red line is slope for DMARDs; green line is for RTX + DMARDs; blue line is for TCZ + DMARDs. Each dot represents one patient. Abbreviations: DMARDs: disease-modifying anti-rheumatic drugs; RTX: Rituximab; TCZ: Tocilizumab.


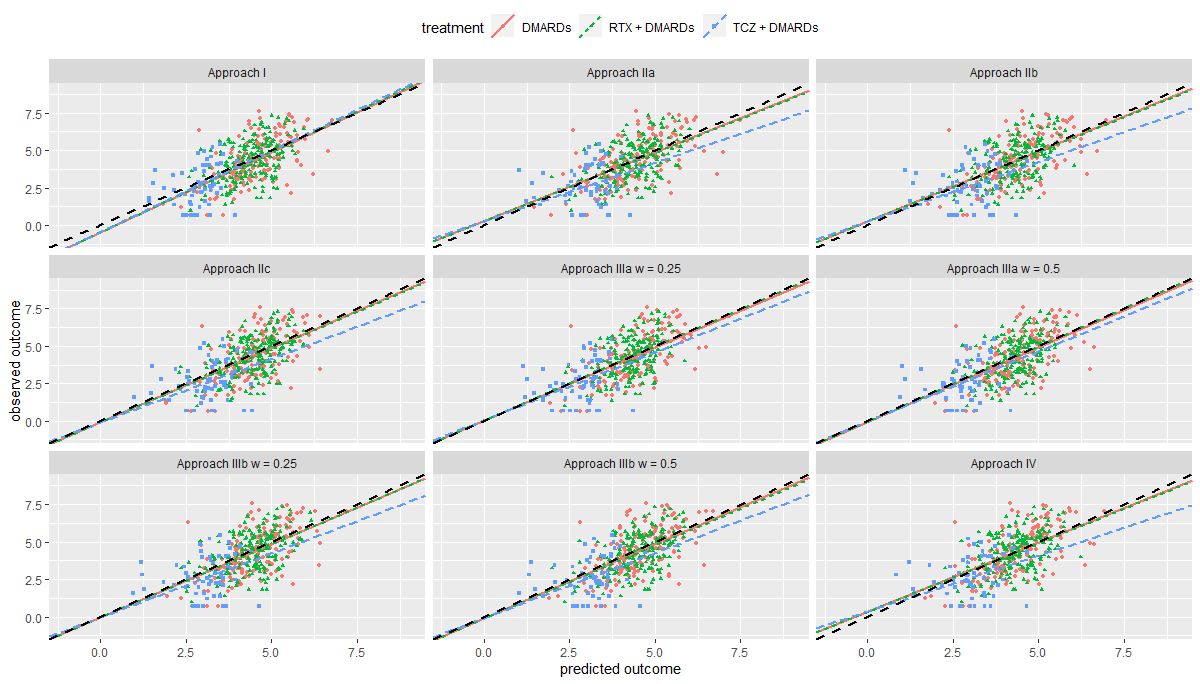


**Figure 2**: Calibration plot from internal validation, for the Swiss registry as the external dataset. Black line is line of perfect calibration. Red line is slope for DMARDs; green line is for RTX + DMARDs; blue line is for TCZ + DMARDs. Each dot represents one patient. Abbreviations: DMARDs: disease-modifying anti-rheumatic drugs; RTX: Rituximab; TCZ: Tocilizumab.


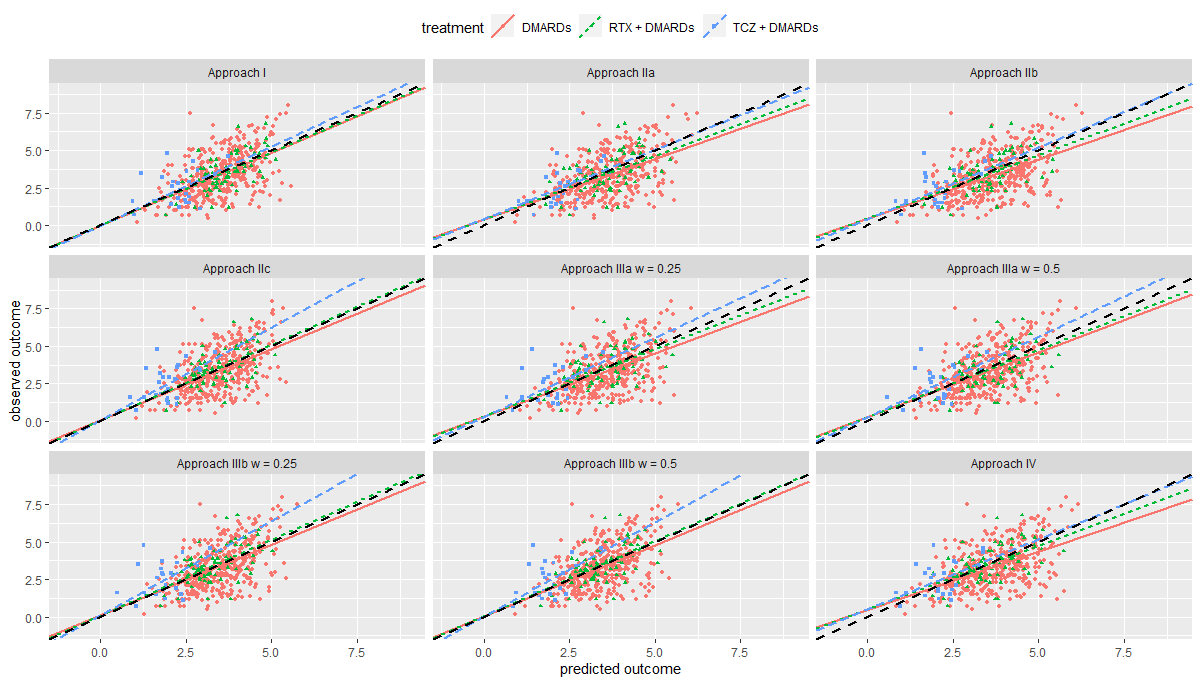

Supplement: sj-docx-1-smm-10.1177_09622802221090759 - Supplemental material for Combining individual patient data from randomized and non-randomized studies to predict real-world effectiveness of interventions [file sj-docx-1-smm-10.1177_09622802221090759.docx]
